# Codon usage patterns in Chinese bayberry (*Myrica rubra*) based on RNA-Seq data

**DOI:** 10.1186/1471-2164-14-732

**Published:** 2013-10-25

**Authors:** Chao Feng, Chang-jie Xu, Yue Wang, Wen-li Liu, Xue-ren Yin, Xian Li, Ming Chen, Kun-song Chen

**Affiliations:** 1 Laboratory of Fruit Quality Biology / The State Agriculture Ministry Laboratory of Horticultural Plant Growth, Development and Quality Improvement, Zhejiang University, Hangzhou, 310058, China; 2 Department of Bioinformatics / The State Key Laboratory of Plant Physiology and Biochemistry, College of Life Sciences, Zhejiang University, Hangzhou, 310058, China; 3 Department of Mathematics, Zhejiang University, Hangzhou, 310027, China

**Keywords:** RNA-Seq, *Myrica rubra*, Chinese bayberry, Codon usage, Codon pairs, Plant evolution, Gene ontology classification, Translation rate, Gene discovery

## Abstract

**Background:**

Codon usage analysis has been a classical topic for decades and has significances for studies of evolution, mRNA translation, and new gene discovery, etc. While the codon usage varies among different members of the plant kingdom, indicating the necessity for species-specific study, this work has mostly been limited to model organisms. Recently, the development of deep sequencing, especial RNA-Seq, has made it possible to carry out studies in non-model species.

**Result:**

RNA-Seq data of Chinese bayberry was analyzed to investigate the bias of codon usage and codon pairs. High frequency codons (AGG, GCU, AAG and GAU), as well as low frequency ones (NCG and NUA codons) were identified, and 397 high frequency codon pairs were observed. Meanwhile, 26 preferred and 141 avoided neighboring codon pairs were also identified, which showed more significant bias than the same pairs with one or more intervening codons. Codon patterns were also analyzed at the plant kingdom, organism and gene levels. Changes during plant evolution were evident using RSCU (relative synonymous codon usage), which was even more significant than GC_3s_ (GC content of 3^rd^ synonymous codons). Nine GO categories were differentially and independently influenced by CAI (codon adaptation index) or GC_3s_, especially in 'Molecular function’ category. Within a gene, the average CAI increased from 0.720 to 0.785 in the first 50 codons, and then more slowly thereafter. Furthermore, the preferred as well as avoided codons at the position just following the start codon AUG were identified and discussed in relation to the key positions in Kozak sequences.

**Conclusion:**

A comprehensive codon usage Table and number of high-frequency codon pairs were established. Bias in codon usage as well as in neighboring codon pairs was observed, and the significance of this in avoiding DNA mutation, increasing protein production and regulating protein synthesis rate was proposed. Codon usage patterns at three levels were revealed and the significance in plant evolution analysis, gene function classification, and protein translation start site predication were discussed. This work promotes the study of codon biology, and provides some reference for analysis and comprehensive application of RNA-Seq data from other non-model species.

## Background

Triplet codons are central to all biological kingdoms, acting as basic coding units or indispensable recognition components in mRNAs to either code for a particular amino acid or cause initiation or termination of a protein chain. Often the same amino acids are encoded by multiple synonymous codons, ranging from two to six, except Met and Trp [1]. Although synonymous mutations are silent in protein sequences according to the central dogma, synonymous codon bias exists widely within and between genomes [2]. As a result, the study of codon usage patterns is beneficial for a better understanding of molecular biology and evolution, mRNA translation, and design of transgenes, new gene discovery, and other biological applications, and has been investigated over several decades [3-6].

The foundation of codon biology is based on the study of full length ORF (open reading frame) sequences from a range of species such as *Caenorhabditis*, *Drosophila*, *Arabidopsis*[3], *Populus*[7], apple [8] kiwifruit [9], and melon [10], which have been obtained mainly from EST technology in recent decades. To date, with the rapid development of deep sequencing technology, large amounts of sequence data have been generated through genome sequencing or RNA-Seq, providing data for a new focus on codon usage patterns [1,2,4,6]. The extensive sequence research in plants has mainly focused on model plant genomes or millions of EST data, from plants such as *Arabidopis*[3,11,12], rice [12-15], *Populus*[16-19] and citrus [20], etc. Similar studies in non-model plants have been neglected, despite the existence of sequence data assembled from RNA-Seq. Further research and analysis in this area can aid the understanding of breeding of crops.

Chinese bayberry (*Myrica rubra* Sieb. and Zucc.), is an economically important subtropical fruit crop native to Asian countries [21]. The fruit is popular throughout China and overseas for its appealing color, distinctive flavor and various bioactive compounds [22,23]. Physiological studies on this plant have been carried out extensively during the last ten years [24-26], and recent research at the molecular level has also been initiated [27-30], especially in the spatio-temporal expression, transcriptional regulatory and functional verification of genes related to anthocyanin [31-34]. However, there is no report on codon usage patterns in Chinese bayberry.

The RNA-Seq project on Chinese bayberry (Accession: PRJNA77861) has been completed, and the data (Accession: SRX176533) made public in our previous study [27]. In the present work, bayberry codon usage was calculated from full length sequences assembled by RNA-Seq. Furthermore, related patterns were revealed mainly through RSCU (relative synonymous codon usage) and CAI (codon adaptation index) at three levels, i.e., across different groups in the plant kingdom, different genes in Chinese bayberry, and different positions in the genes. These analyses will help us to understand the patterns in Chinese bayberry, to improve the research on codon usage in plant biology, and the potential for application of deep sequencing, especially in non-model plants.

## Results and discussion

### Codon usage in Chinese bayberry

Codon usage analysis in Chinese bayberry was based on 1,066 full-length ORF sequences after layers of filtering of 31,665 mRNAs, which were assembled from our previous RNA-Seq data. The overall codon usage Table was created from 354,551 codons, with each codon, excepting stop codons, represented at least 2,216 times (Additional file 1). This amount of data is larger than those used in studies of *Populus*[7], apple [8] and kiwifruit [9].

The overall GC content of 354,551 codons in the study is 0.477, but it varies in different codon positions, with the highest in GC_1_ (GC content of 1^st^ nucleotide in codon, with value at 0.536), lowest in GC_2_ (GC content of 2^nd^ nucleotide in codon, with value at 0.411), and intermediate in GC_3_ (GC content of 3^rd^ nucleotide in codon, with value at 0.484), which is consistent with observations in other plants, such as citrus [20], apple, woodland strawberry, *Arabidopsis thaliana*, etc. (Additional file 2). The GC_3s_ content (GC content of 3^rd^ synonymous codons) of Chinese bayberry is 0.447, similar to but a little smaller than GC_3_ content, because Met and Trp encoding codons (AUG and UGG, with G for 3^rd^ nucleotide), are included in the calculation of GC_3_ content but not GC_3s_. GC_3s_ content of Chinese bayberry is similar to the range in Eudicotyledons (Additional file 2).

**Figure 1:**
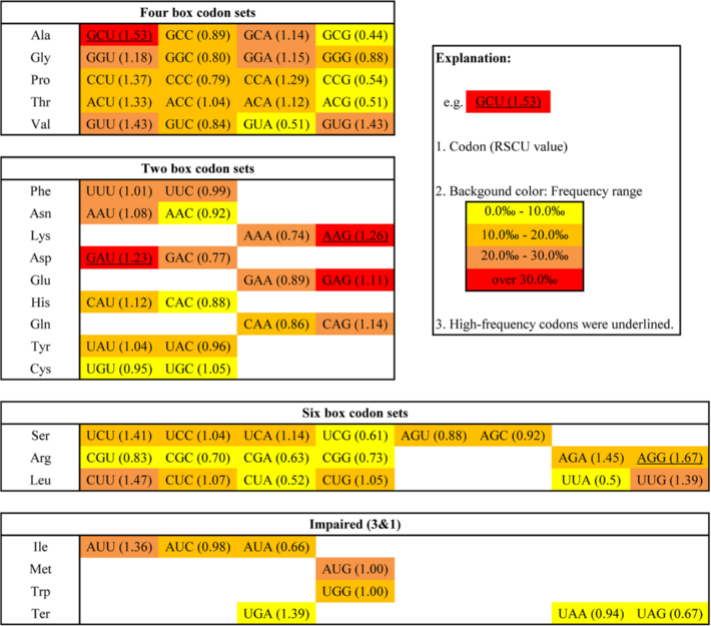
**Unequal enrichment of 64 codons in Chinese bayberry.** The codons for the same amino acids are listed on the same line and are colored yellow, orange yellow, orange and red to reflect the occurrence frequency 0.0‰ - 10.0‰, 10.0‰ - 20.0‰, 20.0‰ -30.0‰, and over 40.0‰, respectively. The data is shown as triplet codon (RSCU, relative synonymous codon usage), and high frequency codons are underlined.

Among 64 codons, AAG was the most frequently presented codon, with the frequency of occurrence 39.8‰, 2.56 times the average frequency. Following this, GAG, GAU and GCU were the next three highest frequency codons, all over 30‰. CGA was the lowest frequency codon, with the frequency of occurrence 5.6‰, only one third of the average, and another 13 codons also had low frequency (below 10‰) (Figure 1).

The RSCU of 64 codons were calculated. AGG and GCU, encoding Arg and Ala, had the highest values (1.67 and 1.53, respectively). AAG and GAU, encoding Lys and Asp, were used more frequently than the synonymous codon for the corresponding amino acids (63.0% and 61.5%, respectively). These four codons have been named high-frequency codons in our study (Figure 1).

Four NCG codons in Chinese bayberry had quite low RSCU (0.44, 0.51, 0.54 and 0.61), which is beneficial for avoiding possible mutation caused by DNA methylation. Because methylated cytosine (C) in the CG dinucleotide is more easily deaminated into thymine (T), and the G in the 3^rd^ codon position is wobbly, therefore the species with a high level of DNA methylation have a tendency to avoid NCG codons to avoid mutation [7,10]. The low RSCU of NCG codons indicate that Chinese bayberry may be a species with a relative high methylation level, which is confirmed by the NCG:NCC ratio. This index has been widely used to estimate CpG suppression, and to reflect the methylation level in mRNA coding sequences, especially in Eudicotyledons, such as *Populus*[7], apple [8], kiwifruit [9] and melon [10]. Species with a low methylation level have a relatively higher NCG:NCC ratio, such as *Arabidopsis thaliana* (0.921), *A. lyrata* (0.93), whereas those with a high methylation level have a relatively lower value, such as grape (0.414), *Populus* (0.463), while intermediate methylation species have intermediate values, such as apple (0.639), tomato (0.634) (Additional file 2). Chinese bayberry has a relatively low NCG:NCC ratio (0.552), which suggests that it is an organism with a relatively higher methylation level. Moreover, a recent report showed that methylation changes have a regulatory effect on tomato ripening [35], so methylation may also play an important regulatory role in Chinese bayberry.

Four NUA codons also have low RSCU (0.5, 0.51, 0.52, and 0.66) (Figure 1), which was also observed in other plant species. This phenomenon can be explained by the hypothesis that reducing UA may increase protein production via inhibition of mRNA degradation [36].

As to stop codons, UGA was the most frequently used, with RSCU of 1.39, UAG was the least used stop codon with the RSCU 0.67, and UAA was intermediate with the RSCU 0.94, which coincided with the overall rules discerned for plants [37].

### Codon pairs in Chinese bayberry

**Figure 2:**
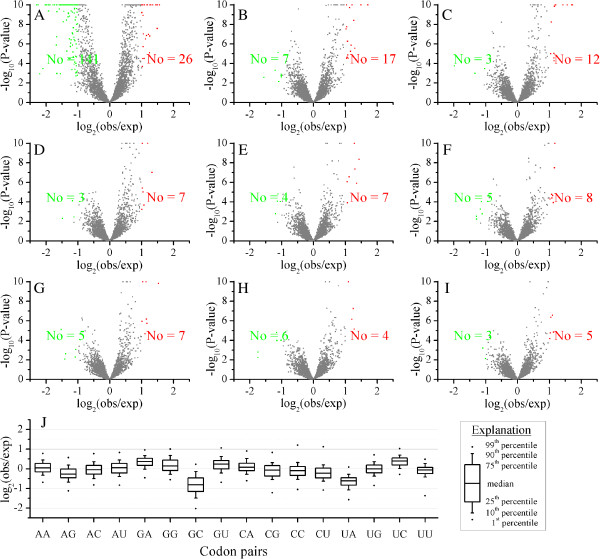
**Overview of preferred and avoided codon pairs in Chinese bayberry. A-I)** The relationship between observed frequency and expected frequency of 3,721 (61*61) codon pairs (excluding stop codons). The red, green and gray spots represent the preferred codon pairs (with P-value less than 0.01, and the ratio of observed value to expected value larger than 2), avoided codon pairs (with P-value less than 0.01, and the ratio of observed value to expected value smaller than 0.5) and unbiased codon pairs, respectively. The lowest P-value was set as 1E^-10^. **A)** The ratio of observed frequency to expected frequency for neighboring codon pairs. The ratio of observed frequency to expected frequency for codon pairs separated by one **(B)**, two **(C)**, three **(D)**, four **(E)**, five **(F)**, six **(G)**, seven **(H)**, eight **(I)** intervening codons. **J)** Distribution of the ratio of observed frequency to expected frequency for different neighboring codon pairs. The X axis shows the last nucleotide of the former codon and the first nucleotide of following codon.

Not only were the synonymous codon in Chinese bayberry not used equally, but codon pairs also showed some bias between observed and expected frequency. When studying neighboring codon pairs, 26 preferred codon pairs and 141 avoided codon pairs were observed (Figure 2A). This bias decreased rapidly with one or two intervening codons between the pairs (Figure 2B,C), and there was almost no bias with codon pairs separated by three to eight intervening codons (Figure 2D - I, Additional file 3). This is consistent with the notion that neighboring codon pair bias may have significance for elucidating the mechanisms for protein synthesis [38].

Moreover, among 141 avoided neighboring codon pairs, 88 pairs (62.4%) showed a pattern where the former codon ended with C and the later codon started with G (Additional file 3), which may relate to a relatively higher methylation level of bayberry DNA, as mentioned above. On the other hand, 37 pairs (26.2%) had UA at the junction (Additional file 3), which may also increase the rate of protein production [36]. These two types were also underrepresented compared to others in overall neighboring codon pairs (Figure 2J), as were mononucleotide repeats, GGGGGG, CCCCCC, UUUUUU. The list of avoided codon pairs in Chinese bayberry is a little different from other species studied [39,40] and could play an important role in transgene design of exogenous genes, especially for Ser, Arg, Leu, Phe and Gly codons.

**Figure 3:**
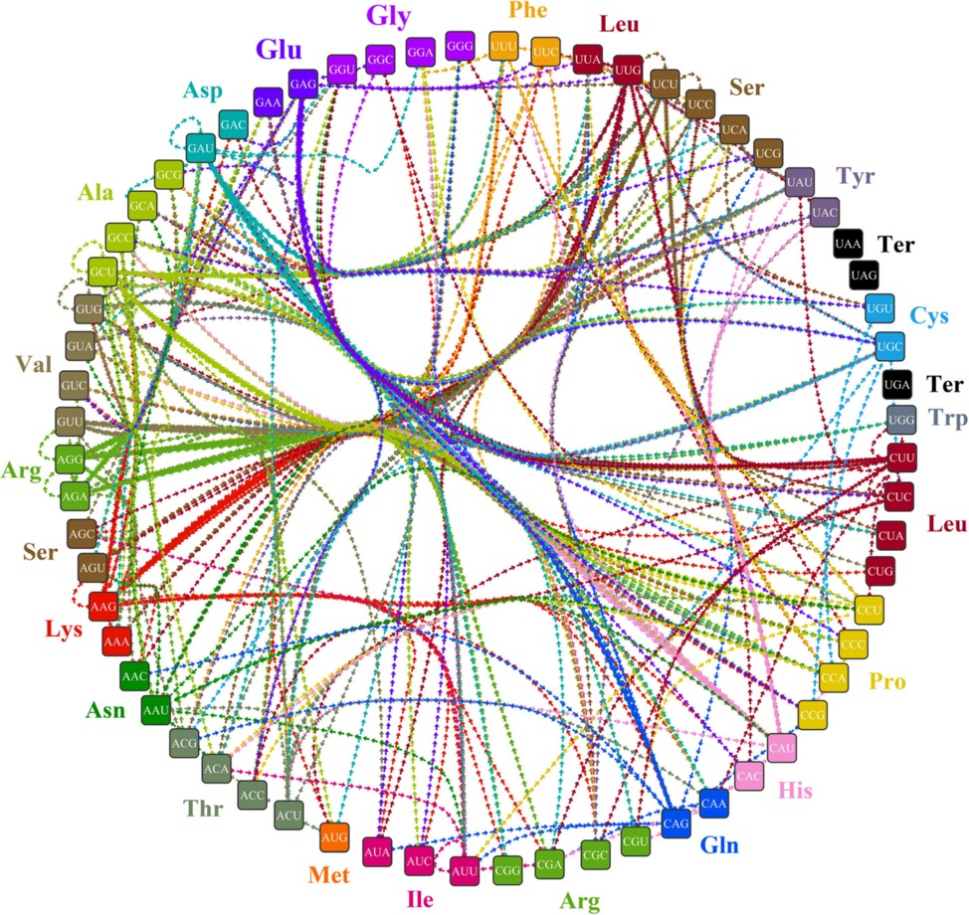
**Overview of high-frequency codon pairs in Chinese bayberry.** 64 codons were put in a clockwise order of 'U’, 'C’, 'A’ and 'G’. Codons encoding the same amino acid or stop codons, as well as the amino acid, are indicated with the same color. The arrows represent high-frequency codon pairs; the direction of arrow is from the preceding codon to the following one, and the arrow colors are the same as for the following codons.

Of 26 preferred neighboring codon pairs, 10 were simple codon repeats (CCGCCG, UCCUCC, UCGUCG, UGCUGC, ACCACC, ACGACG, AGCAGC, CGCCGC, GCGGCG, GGUGGU) (Additional file 3). When a former tRNA was still linked to mRNA, the concentration of the same tRNA in the free state in a cell would decrease; therefore it would take more time for mRNA to obtain the same tRNA, and may play an important role in slowing down the rate of translation of the corresponding mRNA region. The preferred codon pairs are also different to other species [39,40] and may provide a new case for the study of codon biology research. As some bias was shown in neighboring codon pairs (141 avoided and 26 preferred codon pairs), the four high frequency codons (AGG, GCU, AAG and GAU) mentioned above may not always be more significantly highly used than other corresponding synonymous codon when following some specific codons, on the contrary, some other codons may be over-represented when following some other specific codons. Based on the occurrence of codon pairs, 397 high-frequency codon pairs were identified, among which, AAG (Lys), AGG (Arg), GCU (Ala) and GAU (Asp) were found to be the top four likely to be found as the second codon in a pair. They behaved as high frequency codons when following specific 40, 37, 32 and 32 codons respectively. AGA (Arg, 22), GAG (Glu, 20), UUG (Leu, 19), UCU (Ser, 19), CAU (His, 17) ranked as the 5th to 9th highest frequency codons found as the second in a pair of codons. Only 35 codons, rather than all 61 codons, appeared as the second codon in high-frequency codon pairs. However, these 35 codons encode all amino acids with synonymous codons (Figure 3, Additional file 4).

It is widely reported that transforming synonymous codons can significantly influence translational efficiency [2,41,42] and, indeed, a recent case in tomato showed that codon optimization of the *MIR* gene could enhance its expression [43]. Thus, the large-scale identification of high-frequency codons in Chinese bayberry, especially the high-frequency codon pairs (Additional file 4), could be used as a reference in design of exogenous transgenes. Moreover, codon optimization based on the frequency of codon pairs, rather than just high frequency codons, may further promote translational efficiency.

### Codon usage patterns across the plant kingdom

In recent years, many plant genome sequencing projects have been completed and JGI (DOE Joint Genome Institute, version 9), the biggest integrated genome data platform, has a uniform analysis and storage format. In this study, the annotation data of 26 plants, consisting of 5 Chlorophytes (Algae), 1 Bryophyte, 1 Pteridophyte, 5 Monocotyledons and 14 Eudicotyledons, were downloaded and used for codon analysis.

For each plant genome, thousands of full-length ORFs (5,986 to 64,902) and millions of synonymous codons (2,587,991 to 27,829,277) were obtained, and the corresponding GC_1_, GC_2_ and GC_3_ contents were calculated (Additional file 2). In all 27 species (including Chinese bayberry), GC_1_ content was much larger than GC_2_ content, with difference value between 0.096 (*Medicago truncatula*) and 0.155 (*Micromonas pusilla* RCC299). GC_3_ content was a little higher than GC_1_ content in the Gymnosperm, Monocotyledons and Chlorophyte species in this study, while GC_3_ content was similar to GC_2_ content in *Physcomitrella patens* and 15 Eudicotyledons. This indicated that some pressure existed to select G/C in position 1, T/A in position 2, with significant wide variation in position 3.

**Figure 4:**
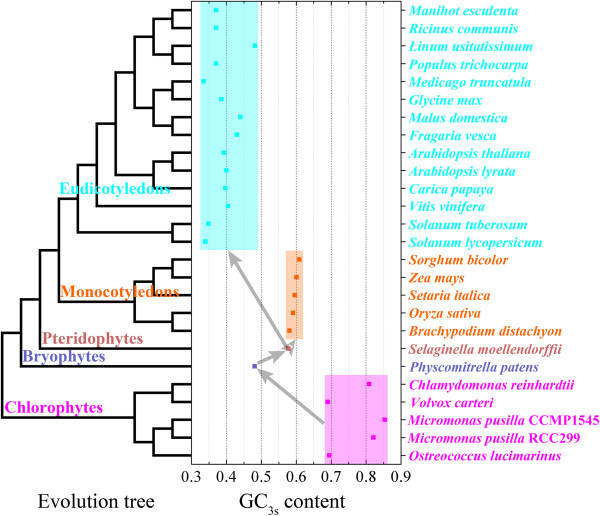
**GC**_**3s **_**distribution in ORFs from 26 plant genomes.** The species belong to Chlorophytes, Bryophytes, Pteridophytes, Monocotyledons and Eudicotyledons are colored magenta, reddish-brown, purple, orange and cyan, respectively. The plant evolutionary tree (left) was drawn according to the JGI homepage (http://www.phytozome.net/). The GC_3s_ distribution in mRNA coding sequences of 26 plants is shown in the center, and the arrows represent the evolutionary transitions from Chlorophytes to Angiosperms (Eudicotyledons and Monocotyledons), via Bryophytes and Pteridophytes.

GC_3s_ varied greatly in different species, and has changed during evolution [44,45], which is also confirmed by our observations (Figure 4). The original single-cell or multiple-cell Chlorophyte plants have very high GC_3s_ contents (0.69 to 0.854), whereas in Bryophytes, GC_3s_ contents was much lower (only 0.481 in *Physcomitrella patens*), and in *Selaginella moellendorffii*, it was 0.578. Evolution of the Angiosperms was accompanied by further variation, and the GC_3s_ contents of Monocotyledons increased (values from 0.581 to 0.609) whereas in the Eudicotyledons, it decreased, with the values between 0.335 and 0.482 (Figure 4). This pattern might be a result of mutation bias, natural selection, or bias in gene conversion across hundreds of million years, as has been observed in yeasts and mammalian [44-46].

**Figure 5:**
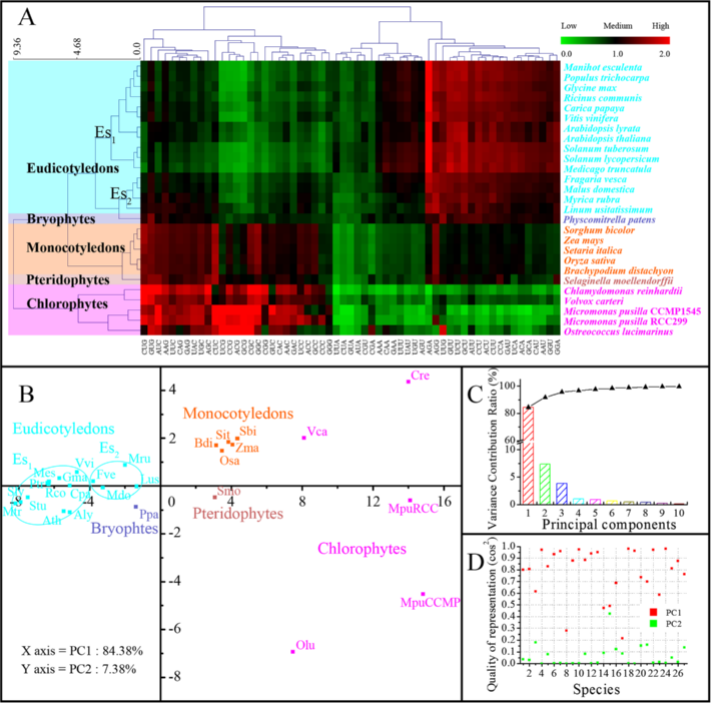
**Heat map and PCA analysis of RSCU of synonymous codons from Chinese bayberry and 26 other plants.** The magenta, reddish-brown, purple, orange and cyan spots represent the Chlorophytes, Bryophytes, Pteridophytes, Monocotyledons and Eudicotyledons, respectively. **A)** Heat map of RSCU of 59 codons from 27 species using Euclidean distance and complete linkage clustering module. **B)** The PCA analysis of RSCU of 59 codons from 27 species on the primary and secondary axes (accounting for 84.38% and 7.38% of the total variation, respectively). **C)** Variance contribution ratio and accumulated variance contribution ratio of the first ten principal components of Part B. **D)** Quality of representation of the first two principal components of Part B, the red and green spots represent the quality of representation of PC1 and PC2, respectively.

Using a single criterion (GC_3s_), can reflect overall plant evolution, but is not ideal at the lower taxonomical level, for example, among three species from the phylum 'Chlorophytes’, *Chlamydomonas reinhardtii*, *Volvox carteri* and *Micromonas pusilla*, the first two are closer, both being in the order 'Volvocales’ of class 'Chlorophyceae’, while *M. pusilla* belongs to class 'Prasinophyceae’, however, GC_3s_ of *C. reinhardtii* is closer to that of *M. pusilla*, rather than *V. carteri*. Therefore, more dimensions were introduced to this study. Comparison of RSCU for all 59 synonymous codons (excluding the Met, Trp, and three stop codons) generated a heat map for Chinese bayberry and 26 plant species by bi-clustering. The results indicated how codon usage bias has changed during plant evolution. Chlorophytes and higher plants are clustered into two primary groups, and higher plants clustered into two sub-branches (Monocotyledons and Eudicotyledons) (Figure 5A). Furthermore, in the Chlorophyte branch and Monocotyledon sub-branch, the clustering trees exactly coincided with the evolution trees of corresponding species (see JGI homepage, http://www.phytozome.net/). In the Eudicotyledons sub-branch, the species from the same genus or closely related genera were clustered into small groups, such as *Arabidopsis thaliana* and *A. lyrata*, *Solanum lycopersicum* (tomato) and *S. tuberosum* (potato), *Fragaria vesca* (woodland strawberry) and *Malus domestica* (apple), etc. (Figure 5A). The codon usage of Chinese bayberry was closest to that of *Linum usitatissimum* (flax) and *Rosaceae* fruit tree (Figure 5A). Moreover, the PCA (principal component analysis) analysis also showed that these 27 species can be divided into 5 groups, the Chlorophytes, Bryophytes, Pteridophytes, Monocotyledons and Eudicotyledons, and Eudicotyledons can also be divided into two subgroups (Figure 5B), coincided with the data revealed by the heat map (Figure 5A). Although 5 Chlorophytes could not be clustered closely to each other, as a result of experiencing a much longer time of variation as the most original plants, they can still be separated from other plants in the first principal component (Figure 5B). In this analysis, the first two axes account for 84.38% and 7.38% of the variation, respectively, and quality of representation is also quite high (Figure 5C, D). Therefore, RSCU, rather than GC_3s_, was a better indicator for evaluating the evolutionary relationships in plants.

Of course, this evaluation indicator needs further development, for *Physcomitrella patens* (Bryophytes) and *Selaginella moellendorffii* (Pteridophytes) have similar GC_3s_ values with *Linum usitatissimum* (Eudicotyledons) and *Brachypodium distachyon* (Monocotyledons), respectively. However, it is possible that the clustering can improve when genome sequencing data for more species of Bryophytes and Pteridophytes are available and can be applied in this analysis (Additional file 2, Figure 5A).

### Codon usage patterns across Chinese bayberry transcripts

Each Chinese bayberry full length ORF sequence was analyzed to discover the patterns of codon usage within a single RNA. Based on RSCU for 59 synonymous codons among 1,066 ORF sequences, correspondence analysis of codon usage (RSCU) and sequences were performed using PCA (Additional file 5). 30 A/U-ending codons and 29 G/C-ending codons were separated into two groups with respect to the first two axes. Meanwhile, 1,066 ORF sequences with different GC_3s_ content could also be separated mainly along the first axis. This result indicates some correlation between codon usage and GC_3s_ among Chinese bayberry ORF sequences, and similar results have also been reported in other plants, such as rice [13]. However, the percentage of contribution of the axes is somewhat low, and may be improved if the genome sequences of Chinese bayberry are available in future.

**Figure 6:**
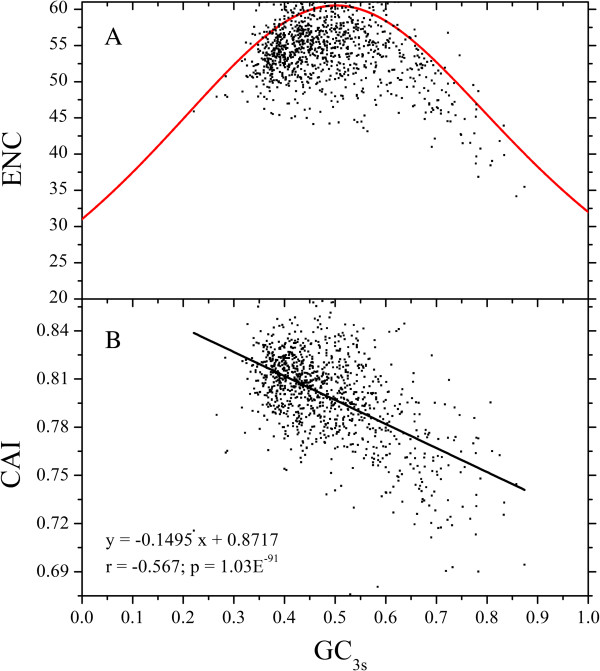
**ENC or CAI versus GC**_**3s **_**plot for 1,066 full length ORFs of Chinese bayberry. A)** ENC versus GC_3s_ plot, the solid red line indicates the expected ENC. **B)** The correlation analysis between CAI and GC_3s_.

To further understand the correlation between codon usage and GC_3s_, effective number of codons (ENC) of 1,066 ORFs was calculated, with the mean value of 54, minimum value of 34.18 and maximum value of 61. Over 88% of 1,066 ORFs have an ENC value lower than the expected one when calculated according to GC_3s_ (Figure 6A), suggesting that mutation pressure is a major factor contributing for the variation of codon usage, and selections play roles during the process of evolution.

Moreover, CAI, another important index of codon usage bias, was introduced to estimate synonymous codon usage bias for each ORF sequence, and a strong negative correlation (r = -0.567, p = 1.03E^-91^) between CAI and GC_3s_ was observed (Figure 6B).

**Figure 7:**
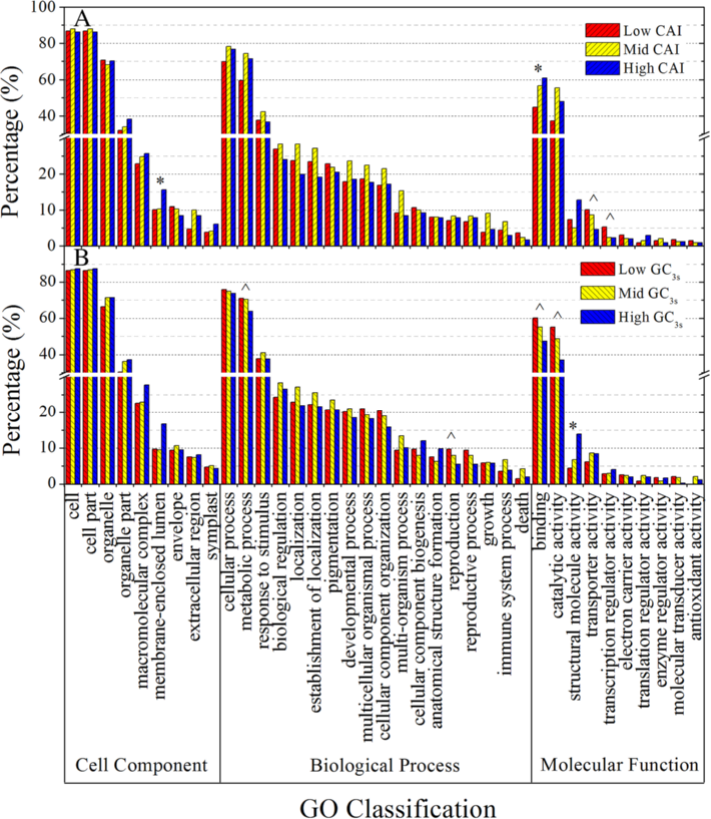
**GO classification for three groups of ORFs.** '*’ indicates in a particular GO category, significantly, at the 5% level, higher percentage of genes in high CAI/GC_3s_ groups than low CAI/GC_3s_ groups, while the percentage of mid CAI/GC_3s_ groups is intermediate. '^’ represents a similar situation, but with the percentage decreasing. **A)** GO classification for three groups of ORFs according to CAI variation. **B)** GO classification for three groups of ORFs according to GC_3s_ variation.

For a better understanding of the influence of either CAI or GC_3s_ for gene ontology (GO) classification, 1,066 ORFs were sorted into 3 groups according to CAI, containing 355, 356 and 355 sequences, among which, 338, 339 and 346 had GO annotation. Similarly, another three groups (with 355, 356 and 355 ORFs) were generated according to GC_3s_ value, with 340, 338 and 345 ORFs in the corresponding groups having GO annotation. GO classifications were performed individually for groups sorted according to values of either CAI or GC_3s_. Categories with significant difference among three groups were observed in 'Molecular Function’. The 'binding’ Category has the highest representation of ORFs, and the percentage of ORF related to this category increased as CAI increased while it increased as GC_3s_ decreased, suggesting that the genes belonging to 'Binding’ Category were positively influenced by CAI, but negatively influenced by GC_3s_. These genes, lower GC_3s_ in total, may be under a higher degree of positive selection of codon bias, which can result in a tendency of a higher translate rate. The other four large categories in 'Molecular Function’ also showed differential representation among either CAI or GC_3s_ groups. Enrichment of high CAI group was observed in the 'membrane-enclosed lumen part’ Category, and there was a downward trend of the high GC_3s_ group in 'metabolic processes’ and 'reproduction’ Category (Figure 7). In total, nine GO categories were differentially and independently influenced by CAI (codon adaptation index) or GC_3s_, suggesting that these categories may have special codon usage bias or GC_3s_ bias, and CAI as well as GC_3s_ may be related to gene function.

### Codon usage patterns across different positions in each gene

**Figure 8:**
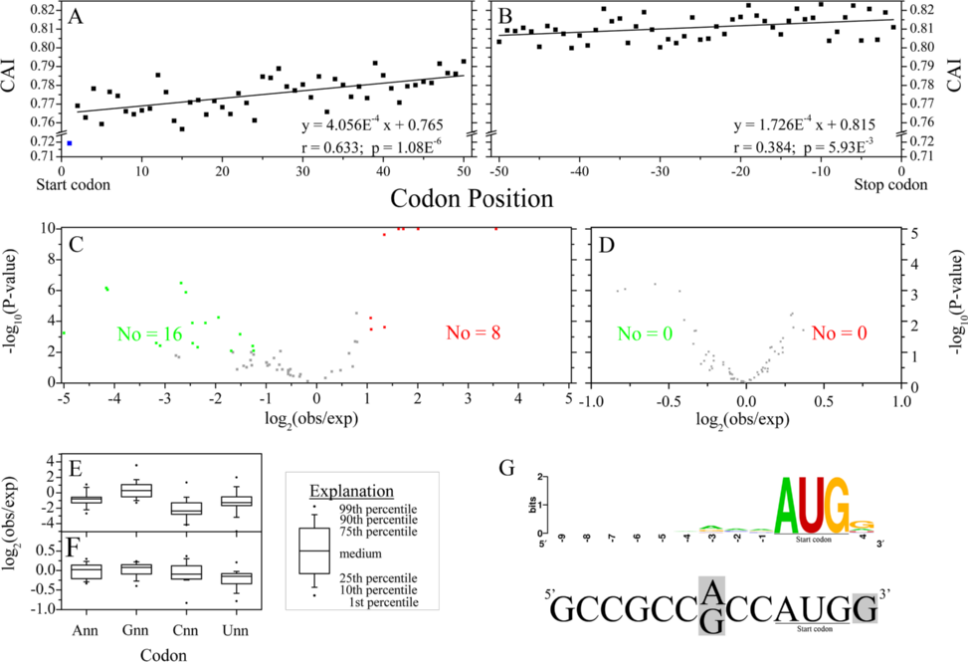
**Overview of codon usage and the preferred and avoided codons following AUG in Chinese bayberry. A)** Correlation between CAI and codon positions of the first 50 codons following the start codon on a scatter diagram, with linear fitting of 2–50 codons. **B)** Correlation between CAI and codon positions of the last 50 codons before stop codons, on a scatter diagram, with linear fitting. **C, D)** The relationship between observed frequency and expected frequency of 61 codons following the start codon AUG **(C)** and non-start internal codon AUG **(D)**. The red, green and gray spots represent the preferred codons (with P-value less than 0.01 and the ratio of observed value to expected value larger than 2, avoided codons (with P-value less than 0.01, and the ratio of observed value to expected value smaller than 0.5) and unbiased codon pairs, respectively. The lowest P-value was set as 1E^-10^. **E, F)** Distribution of the ratio of observed frequency to expected frequency in different types of codons following the start codon AUG **(E)** and non-start internal codon AUG **(F)**. The X axis shows the types of codons, where n represent A, G, C or U. **G)** Comparisons of bayberry and Kozak consensus sequences of 13 nucleotides (n9AUGn) around the start codon AUG. The upper, colored part shows the consensus sequence of 13 nucleotides (n9AUGn) around the start codon AUG in Chinese bayberry. The lower part is the Kozak consensus sequence of 13 nucleotides (n9AUGn) around the start codon AUG, and the nucleotides with gray background indicate the key recognition sites.

Codon usage varied dramatically within a single gene. CAI increased from the beginning to the end of the ORF, particularly over the first part of the ORF. For example, the CAI increased from 0.720 to 0.785 over the first 50 codons (r = 0.633, p = 1.08E^-6^) (Figure 8). It is well known that favored codons are usually associated with highly abundant tRNAs and tend to be translated much faster [47]. On the contrary, rare codons are recognized by low abundance tRNAs and are likely to be translated relatively more slowly. Thus, the higher CAI is related to higher translate rate. The data suggest that the translation rate would be low after translation initiation, and increased quickly over the first part of the ORF, and then slowly thereafter. In eukaryotes, several ribosomes can bind to an mRNA molecule at the same time, as polysomes, and multiple protein molecules can be synthesized from a single mRNA at the same time. The increase in CAI along the beginning of ORFs ensures may be an adaptation to the multi-polysome based translation process.

The average CAI of the codon following the start codon in all the 1,066 ORFs was 0.720, much smaller than other positions (Figure 8A). In further analysis, the nucleotides (or codons) following the start codon or non-start codon AUG were compared to understand the significance of this phenomenon (Figure 8C - F). It was found that 'G’ is the preferred nucleotide following the start codon AUG (Figure 8E). This 'G’, together with 'A/G’ just preceding the start codon AUG, are the key positions in the Kozak sequence for identification of the translation start site (Figure 8G) [48], while a 'C’ following the start codon AUG tends to be less favored (Figure 8E) as our data confirms, with 15 of 16 CNN codons at this position under-represented compared to other positions. However, CCG represented 2.5 fold of expected frequency. Besides, the three other NCG codons are also over-represented (1.63, 4.03 and 11.79 fold) following the start codon AUG. On the whole, the NCG:NCC ratio is 2.23, four fold of the overall ratio, suggesting that this site maybe one of the most important for methylation regulation in this species. Meanwhile, 8 preferred and 16 avoided codons were observed for the codon following the start codon AUG (Figure 8C). CCG, one of the 8 preferred codons, as mentioned above, having the lowest expected frequency among codons encoding Pro, was over-abundant, while the other three codons encoding Pro occurred less frequently than expected. Similarly, two out of six Ser codons with low expected frequency were over-represented as well, while two out of three Ile codons with high expected frequency were under-represented. At the amino acid level, it was found that Ala was the most preferred amino acid following the initiating Met, while many other amino acids, such as Cys, Ile, Leu, His, Met and Trp, tended to be less favored (Additional file 3). In contrast, no bias was observed in the codons following internal AUG codons (Figure 8D). This finding could be further used for construction of an algorithm to predict the TSS (translation start site) of a gene, and therefore could aid gene characterization in Chinese bayberry, and provide a reference for other species.

## Conclusions

A comprehensive codon usage Table in Chinese bayberry was established and the numbers of high-frequency codon pairs were analyzed. Underrepresentation of codons NCG and NUA was observed, which may have the function of avoiding the mutation caused by DNA methylation and increasing protein production, respectively. Prominent bias on neighboring codon pairs was also found, indicating possible mechanistic significance in regulating protein synthesis rate. Codon usage patterns were comprehensively analyzed at plant kingdom, organism, and gene levels. It was found that RSCU is strongly related to plant evolution, and is even more significant than GC_3s_. At the species level, nine GO categories were differentially and independently influenced by CAI (codon adaptation index) or GC_3s_. Within a gene, CAI increased from the beginning to the end of an ORF, especially during the first 50 codons, which may be beneficial for translation efficiency. The codons following the start codon AUG have the lowest CAI and greatest bias, which is related to a special translation start recognition motif of Kozak sequence (*A/G*NNAUG*G*). This feature may play an important role in prediction of the translation start site and discovery of new genes (or transcripts). These findings established knowledge of the codon patterns of Chinese bayberry, provided additional information for the study of codon biology, and a reference for comprehensive analysis and application of RNA-Seq data to other non-model species.

## Methods

### Sequence data collection, filtering and mining

The dataset is comprised of two main parts, firstly, RNA-Seq data of Chinese bayberry which was downloaded from NCBI SRA (Sequence Read Archive) database (http://www.ncbi.nlm.nih.gov/Traces/sra/, Accession No.: SRX176533), and further assembly and annotation as in our previous work [27]. Secondly, protein-coding sequences (*_cds.fa.gz and *_protein.fa.gz) from 26 of the published plant genomes were downloaded from JGI (ftp://ftp.jgi-psf.org/pub/compgen/phytozome/v9.0) on Jan 16^th^, 2013.

Full length coding sequences were identified, beginning with an AUG start codon, ending with UAA, UAG or UGA stop codon. From these, low quality sequences, i.e., sequences of a length no more than 300 bp, or those having an internal stop codon, were excluded. Then additional filtering steps were used to remove low quality sequences mined from bayberry RNA-Seq. The sequences containing uncertain nucleotides or encoding low abundance genes, those with RPKM (reads per kb per million reads) value lower than 10, and those obviously incomplete or too long (less than 95% or over 105% when compared to the length of top hit homologous sequences from other plants using BLASTx with an e-value cutoff of 1e^-5^) were excluded. All the above procedures were performed with Microsoft Excel 2010 and some PERL scripts written in-house.

The first 50 codons (excluding the start codon) and the last 50 codons (excluding the stop codon) in each gene obtained via filtering were named as cod_1 to cod_50, and cod_-1 to cod_-50, respectively. Codons with the same name were mixed, stored in Fasta format, and then the new rearranged sequences were structured to represent the situation of codon distribution at different positions. All the above procedures were performed using Microsoft Excel 2010 and PERL scripts written in-house.

After filtering and rearranging, sequences were used to calculate the basic index of codon usage, such as the nucleotide composition at the 3^rd^ codon position, the codon number, RSCU, and ENC, using codonW 1.4.2 (http://codonw.sourceforge.net). RSCU is calculated according to the formula described in Sharp and Li [49]. Codons with RSCU over 1.0 occur at high frequency and the larger the number the more significant the bias, while numbers below 1.0 indicates the opposite.

ENC is calculated according to the formula described in Wright [50], and it is a measure of the unevenness of use of codons for all the 20 amino acids across ORFs, with the value between 20 and 61. The value is 20 when only one special codon is used for each amino acid, while it is 61 when all the codons are equally used for each amino acid.

CAI is calculated using the formula described in Sharp and Li [51], and is widely applied in estimating codon usage bias, with the value between 0 and 1. The larger the value the greater the degree of positive selection, while the lower the value the greater the degree of negative bias.

### Identification of high-frequency codons (or codon pairs)

From the calculation of RSCU of all the full length protein-coding sequences, codons with RSCU over 1.5, or those having a relative frequency above 60% of synonymous codon for the corresponding amino acids, were selected and defined as high-frequency codons [19,52]. The concept of high-frequency codon pairs is as follows. For neighboring amino acids, codon X is the first amino acid, and candidate codons Y_1_ to Y_i_ is the second amino acid, where i is equal to the number of synonymous codons for the second amino acid. If the occurrence of neighboring Codon XY_j_ (1 ≤ j ≤ i) is over 1.5 fold above the average occurrence of neighboring Codon XY_1_ to XY_i_, or takes over 60% of total occurrence of neighboring Codon XY_1_ to XY_i_, calculating from data from full length ORFs, excluding the first and stop codons, then Codon XY_j_ is called a high-frequency codon pair. Identification of the high-frequency codon pairs was performed using PERL scripts written in-house and the software Cytoscape (version 3.0.1, http://www.cytoscape.org/) [53].

### Identification of preferred and avoided codons (or codon pairs)

The expected frequency is the ratio of the total occurrence of a certain codon to the total occurrence of all 61 codons (excluding the stop codons and the AUG when serving as the start codon), calculating from the ORFs (excluding the first and the last codons). The observed frequency is the ratio of the actual occurrence of a certain codon in a certain position of all ORFs to the total occurrence of all 61 codons in that position. The frequencies of the codons following the start codon AUG, and those following non-start internal AUG codon were also calculated. Frequencies with p-values less than 0.01 were considered statistically significant, and the ratio of observed frequency to expected frequency (log_2_), with a ratio cutoff of ±1 (2 fold changes), was the standard used to identify the preferred or avoided codon pairs. P-value was calculated following the formula described in Audic and Claverie [54] via our previous PERL program [27].

The expected frequency of 3721 (61*61) codon pairs (both the neighboring codon pairs and those separated by several intervening codons) is the product of the corresponding expected frequencies of each codon. The observed frequency of codon pairs is the ratio of occurrence of a certain pair to occurrence of all 3721 codon pairs, calculating from full length ORFs excluding the first and stop codons, as mentioned above. The parameters for screening preferred and avoided codon pairs were the same as described above. All procedures were performed using Microsoft Excel 2010 and PERL scripts written in-house.

### Cluster and PCA analysis

The RSCU of 59 codons within synonyms from Chinese bayberry and 26 other plants were calculated by complete linkage clustering with Euclidean distance using Mev v4.8.1 [55] (http://sourceforge.net/projects/mev-tm4/files/mev-tm4/) software. PCA of these 27 plants were performed based on RSCU of 59 synonymous codons, meanwhile variance contribution ratio, accumulated variance contribution ratio and quality of representation (cos^2^_α_, α is the angle of spot vector and axial vector) were calculated using MATLAB (version 7.0) and drawn by OriginLab Origin (version 8.0, Microcal Software Inc., Northampton, MA, USA). RSCU of 59 synonymous codons from all the bayberry full length ORFs (59 spots) were reduced from 1,066 dimensions (1,066 ORFs) into two principal components by PCA method, while for the transposed matrix, all the full length ORFs with RSCU value of 59 synonymous codons (1,066 spots) were reduced from 59 dimensions (59 codons) into two principal components, using the same procedure.

### Gene ontology annotation

Gene Ontology annotation of full length ORFs was performed using Blast2GO (http://www.blast2go.com) [56], and GO classifications were compared among different groups according to CAI or GC_3s_ variation using WEGO (http://wego.genomics.org.cn/cgi-bin/wego/index.pl) [57].

### Identification of 13 consensus nucleotides (n9AUGn)

The 13 nucleotides (including 9 nucleotides before the start codon, 3 nucleotides of the start codon and 1 nucleotide following the start codon) of each mRNAs were picked out via PERL scripts written in-house, where N represents an unknown nucleotide. The consensus motif was created using WEBLOGO (http://weblogo.berkeley.edu) [58].

### Statistical analyses

The correlation analysis between CAI and GC_3s_, ENC and GC_3s_, ENC and CAI, or between CAI and codon position, was performed and drawn by OriginLab Origin (version 8.0, Microcal Software Inc., Northampton, MA, USA) software. Meanwhile, the corresponding parameters, such as linear regression equation, r value, p value, were calculated using MATLAB (version 7.0). Other simple statistical analyses were performed mainly in Microsoft Excel 2010.

## Abbreviations

C: Cytosine; CAI: Codon adaptation index; ENC: Effective number of codons; GC1: GC content of 1st nucleotide in codon; GC2: GC content of 2nd nucleotide in codon; GC3: GC content of 3rd nucleotide in codon; GC3s: GC content at 3rd nucleotide of synonymous codon; GO: Gene ontology; JGI: DOE joint genome institute; ORF: Open reading frame; PCA: Principal component analysis; RPKM: Reads per kb per million reads; RSCU: Relative synonymous codon usage; TSS: Translation start site.

## Competing interests

The authors declare that they have no competing interests.

## Authors’ contributions

CF analyzed the data and drafted the manuscript. CX contributed to the research design and participated in writing the manuscript. YW wrote the PERL scripts. WL, XY, XL and MC participated in data analysis and reviewed the manuscript. KC initiated the project, designed the research framework and coordinated the study. All authors read and approved the final manuscript.

## Supplementary Material

Additional file 1 Codon usage Table in Chinese bayberry. Click here for file

Additional file 2 Basic information for ORFs of 26 plants. Click here for file

Additional file 3 Integrated information of observed and expected frequency of codon pairs, and codons following AUG. Click here for file

Additional file 4 **18 groups of high-frequency codon pairs for codons for 18 amino acids (expecting Met and Trp) in Chinese bayberry.** 64 codons were put in a clockwise order of 'U’, 'C’, 'A’ and 'G’. Codons encoding the same amino acid or stop codons, as well as the amino acid, were indicated with the same color. The arrow lines represent high-frequency codon pairs; the direction of the arrow links the first codon to the one following, and the color is the same as that of the following codons. Click here for file

Additional file 5 **PCA analysis of RSCU of codons within synonym groups from all bayberry ORF sequences. A)** The distribution of 59 codons on the primary and secondary axes (accounting for 29.39% and 10.49% of the total variation, respectively), A/U-ending and G/C-ending codons are colored red and green, respectively. **B)** The distribution of 1,066 ORF sequences on the primary and secondary axes (accounting for 18.19% and 4.34% of the total variation, respectively), different groups of ORFs with GC3s content less than 0.40, 0.40 - 0.45, 0.45 - 0.50, 0.50 - 0.55, 0.55 - 0.65, and over 0.65, are colored red, yellow, green, cyan, blue and magenta, respectively. Click here for file
