# Supplementary material for: Codon usage patterns in Chinese bayberry (Myrica rubra) based on RNA-Seq data
Source: BMC Genomics. 2013 Oct 25;14:732. doi: 10.1186/1471-2164-14-732 (PMC4008310; doi:10.1186/1471-2164-14-732)
Supplement: Additional file 1 — Codon usage Table in Chinese bayberry. [file 1471-2164-14-732-S1.doc]

**Additional file 1.** Codon usage Table in Chinese bayberry

| AA | Codon | NO.^a^ | 1/1k^b^ | RSCU^c^ | AA | Codon | NO.^a^ | 1/1k^b^ | RSCU^c^ | AA | Codon | NO.^a^ | 1/1k^b^ | RSCU^c^ | AA | Codon | NO.^a^ | 1/1k^b^ | RSCU^c^ |
| --- | --- | --- | --- | --- | --- | --- | --- | --- | --- | --- | --- | --- | --- | --- | --- | --- | --- | --- | --- |
| Phe | UUU | 7269 | 20.5 | 1.01 | Ser | UCU | 6450 | 18.2 | 1.41 | Tyr | UAU | 5452 | 15.4 | 1.04 | Cys | UGU | 2710 | 7.6 | 0.95 |
|  | UUC | 7151 | 20.2 | 0.99 |  | UCC | 4743 | 13.4 | 1.04 |  | UAC | 5061 | 14.3 | 0.96 |  | UGC | 2983 | 8.4 | 1.05 |
| Leu | UUA | 2701 | 7.6 | 0.50 |  | UCA | 5238 | 14.8 | 1.14 | Ter | UAA | 334 | 0.9 | 0.94 | Ter | UGA | 493 | 1.4 | 1.39 |
|  | UUG | 7561 | 21.3 | 1.39 |  | UCG | 2792 | 7.9 | 0.61 |  | UAG | 239 | 0.7 | 0.67 | Trp | UGG | 4174 | 11.8 | 1.00 |
|  | CUU | 7945 | 22.4 | 1.47 | Pro | CCU | 6285 | 17.7 | 1.37 | His | CAU | 4270 | 12.0 | 1.12 | Arg | CGU | 2594 | 7.3 | 0.83 |
|  | CUC | 5823 | 16.4 | 1.07 |  | CCC | 3623 | 10.2 | 0.79 |  | CAC | 3382 | 9.5 | 0.88 |  | CGC | 2185 | 6.2 | 0.70 |
|  | CUA | 2829 | 8.0 | 0.52 |  | CCA | 5916 | 16.7 | 1.29 | Gln | CAA | 5490 | 15.5 | 0.86 |  | CGA | 1968 | 5.6 | 0.63 |
|  | CUG | 5676 | 16.0 | 1.05 |  | CCG | 2474 | 7.0 | 0.54 |  | CAG | 7323 | 20.7 | 1.14 |  | CGG | 2277 | 6.4 | 0.73 |
| Ile | AUU | 8487 | 23.9 | 1.36 | Thr | ACU | 5824 | 16.4 | 1.33 | Asn | AAU | 7518 | 21.2 | 1.08 | Ser | AGU | 4047 | 11.4 | 0.88 |
|  | AUC | 6082 | 17.2 | 0.98 |  | ACC | 4555 | 12.8 | 1.04 |  | AAC | 6350 | 17.9 | 0.92 |  | AGC | 4220 | 11.9 | 0.92 |
|  | AUA | 4090 | 11.5 | 0.66 |  | ACA | 4887 | 13.8 | 1.12 | Lys | AAA | 8314 | 23.4 | 0.74 | Arg | AGA | 4538 | 12.8 | 1.45 |
| Met | AUG | 8692 | 24.5 | 1.00 |  | ACG | 2216 | 6.3 | 0.51 |  | AAG | 14128 | 39.8 | 1.26 |  | AGG | 5248 | 14.8 | 1.67 |
| Val | GUU | 9014 | 25.4 | 1.43 | Ala | GCU | 10666 | 30.1 | 1.53 | Asp | GAU | 11561 | 32.6 | 1.23 | Gly | GGU | 7405 | 20.9 | 1.18 |
|  | GUC | 5273 | 14.9 | 0.84 |  | GCC | 6227 | 17.6 | 0.89 |  | GAC | 7170 | 20.2 | 0.77 |  | GGC | 5009 | 14.1 | 0.80 |
|  | GUA | 3218 | 9.1 | 0.51 |  | GCA | 7970 | 22.5 | 1.14 | Glu | GAA | 10203 | 28.8 | 0.89 |  | GGA | 7206 | 20.3 | 1.15 |
|  | GUG | 7626 | 21.5 | 1.21 |  | GCG | 3080 | 8.7 | 0.44 |  | GAG | 12805 | 36.1 | 1.11 |  | GGG | 5511 | 15.5 | 0.88 |

^a^ the number of codons in this set; ^b^ Codon frequency normalized per 1,000 bases; ^c^ RSCU, relative synonymous codon usage.
