# Supplementary material for: Codon usage patterns in Chinese bayberry (Myrica rubra) based on RNA-Seq data
Source: BMC Genomics. 2013 Oct 25;14:732. doi: 10.1186/1471-2164-14-732 (PMC4008310; doi:10.1186/1471-2164-14-732)
Supplement: Additional file 2 — Basic information for ORFs of 26 plants. [file 1471-2164-14-732-S2.doc]

**Additional file 2.** Basic information of ORFs of 26 plants

| Species | Classification | Ref | No. Raw ^a^ | No. Filter ^b^ | GC_1_ ^c^ | GC_2_ ^d^ | GC_3_ ^e^ | NCG/NCC ^f^ | L_sym ^g^ | GC_3s_ ^h^ |
| --- | --- | --- | --- | --- | --- | --- | --- | --- | --- | --- |
| *Arabidopsis lyrata* | Eudicotyledons |  | 32670 | 27271 | 0.504 | 0.402 | 0.422 | 0.930 | 10700903 | 0.4 |
| *Arabidopsis thaliana* | Eudicotyledons |  | 35386 | 32818 | 0.506 | 0.403 | 0.415 | 0.921 | 13771073 | 0.393 |
| *Brachypodium distachyon* | Monocotyledons |  | 31029 | 29508 | 0.570 | 0.441 | 0.596 | 0.791 | 12541560 | 0.581 |
| *Carica papaya* | Eudicotyledons |  | 27760/27775 | 20594 | 0.506 | 0.405 | 0.420 | 0.581 | 7134923 | 0.397 |
| *Chlamydomonas reinhardtii* | Chlorophyte |  | 19526 | 18147 | 0.718 | 0.576 | 0.814 | 1.201 | 13800389 | 0.809 |
| *Fragaria vesca* | Eudicotyledons |  | 32831 | 29969 | 0.516 | 0.412 | 0.451 | 0.613 | 12015825 | 0.43 |
| *Glycine max* | Eudicotyledons |  | 73320 | 64279 | 0.503 | 0.400 | 0.408 | 0.420 | 27361013 | 0.386 |
| *Linum usitatissimum* | Eudicotyledons |  | 4348 | 40885 | 0.522 | 0.417 | 0.501 | 0.781 | 16482743 | 0.482 |
| *Malus domestica* | Eudicotyledons |  | 63517 | 51330 | 0.513 | 0.410 | 0.460 | 0.639 | 20195735 | 0.44 |
| *Manihot esculenta* | Eudicotyledons |  | 34151 | 27447 | 0.506 | 0.405 | 0.394 | 0.441 | 11164558 | 0.371 |
| *Medicago truncatula* | Eudicotyledons |  | 45888 | 33160 | 0.481 | 0.385 | 0.359 | 0.539 | 12070689 | 0.335 |
| *Micromonas pusilla* CCMP1545 | Chlorophyte |  | 10660 | 8943 | 0.658 | 0.509 | 0.927 | 2.937 | 4212681 | 0.854 |
| *Micromonas pusilla* RCC299 | Chlorophyte |  | 10103 | 8852 | 0.644 | 0.489 | 0.852 | 1.449 | 4426739 | 0.821 |
| *Oryza sativa* | Monocotyledons |  | 49061 | 44574 | 0.573 | 0.450 | 0.605 | 0.904 | 16634560 | 0.591 |
| *Ostreococcus lucimarinus* | Chlorophyte |  | 7796 | 5986 | 0.582 | 0.456 | 0.736 | 3.140 | 2587991 | 0.695 |
| *Physcomitrella patens* | Bryophyta |  | 38354 | 30140 | 0.548 | 0.431 | 0.500 | 0.902 | 12393311 | 0.481 |
| *Populus trichocarpa* | Eudicotyledons |  | 73013 | 64902 | 0.507 | 0.405 | 0.394 | 0.463 | 27829277 | 0.371 |
| *Ricinus communis* | Eudicotyledons |  | 31221 | 23597 | 0.509 | 0.406 | 0.394 | 0.573 | 9066981 | 0.371 |
| *Selaginella moellendorffii* | Gymnosperm |  | 22285 | 19298 | 0.560 | 0.423 | 0.594 | 1.078 | 6944592 | 0.578 |
| *Setaria italica* | Monocotyledons |  | 40599 | 31504 | 0.576 | 0.447 | 0.610 | 0.831 | 13265583 | 0.596 |
| *Solanum lycopersicum* | Eudicotyledons |  | 34727 | 28737 | 0.493 | 0.393 | 0.365 | 0.634 | 11066221 | 0.34 |
| *Solanum tuberosum* | Eudicotyledons |  | 51472 | 44376 | 0.497 | 0.398 | 0.374 | 0.590 | 14415158 | 0.349 |
| *Sorghum bicolor* | Monocotyledons |  | 29448 | 26698 | 0.579 | 0.447 | 0.622 | 0.853 | 11240125 | 0.609 |
| *Vitis vinifera* | Eudicotyledons |  | 26346 | 21813 | 0.507 | 0.403 | 0.428 | 0.414 | 8830682 | 0.405 |
| *Volvox carteri* | Chlorophyte |  | 15285 | 12665 | 0.658 | 0.523 | 0.700 | 1.076 | 6972190 | 0.69 |
| *Zea mays* | Monocotyledons |  | 63540 | 53716 | 0.580 | 0.455 | 0.615 | 0.862 | 19227050 | 0.601 |

^a^ the numbers of original sequences from ORF annotation and protein annotation, if this two numbers are the same, just one digit is showed, if not, two digits are showed by turn, like in *Carica papaya*, there are 27760 CDS and 27775 protein as the annotation. ^b^ the number of full length coding sequences after filtering. ^c^ the GC content of 1^st^ codon. ^d^ the GC content of 2^nd^ codon. ^e^ the GC content of 3^rd^ codon. ^f^ the ratio of CG-end codons and CC-end codons. ^g^ the number of synonymous codons. ^h^ the GC content of 3^rd^ synonymous codon.

References：

1. Hu TT, Pattyn P, Bakker EG, Cao J, Cheng JF, Clark RM, Fahlgren N, Fawcett JA, Grimwood J, Gundlach H *et al*: **The *Arabidopsis lyrata* genome sequence and the basis of rapid genome size change**. *Nat Genet* 2011, **43**(5):476-481.

2. Swarbreck D, Wilks C, Lamesch P, Berardini TZ, Garcia-Hernandez M, Foerster H, Li D, Meyer T, Muller R, Ploetz L *et al*: **The Arabidopsis Information Resource (TAIR): gene structure and function annotation**. *Nucleic Acids Res* 2008, **36**(Database issue):D1009-1014.

3. **Genome sequencing and analysis of the model grass *Brachypodium distachyon***. *Nature* 2010, **463**(7282):763-768.

4. Ming R, Hou S, Feng Y, Yu Q, Dionne-Laporte A, Saw JH, Senin P, Wang W, Ly BV, Lewis KL *et al*: **The draft genome of the transgenic tropical fruit tree papaya (*Carica papaya* Linnaeus)**. *Nature* 2008, **452**(7190):991-996.

5. Merchant SS, Prochnik SE, Vallon O, Harris EH, Karpowicz SJ, Witman GB, Terry A, Salamov A, Fritz-Laylin LK, Marechal-Drouard L *et al*: **The Chlamydomonas genome reveals the evolution of key animal and plant functions**. *Science* 2007, **318**(5848):245-250.

6. Shulaev V, Sargent DJ, Crowhurst RN, Mockler TC, Folkerts O, Delcher AL, Jaiswal P, Mockaitis K, Liston A, Mane SP *et al*: **The genome of woodland strawberry (*Fragaria vesca*)**. *Nat Genet* 2011, **43**(2):109-116.

7. Schmutz J, Cannon SB, Schlueter J, Ma J, Mitros T, Nelson W, Hyten DL, Song Q, Thelen JJ, Cheng J *et al*: **Genome sequence of the palaeopolyploid soybean**. *Nature* 2010, **463**(7278):178-183.

8. Wang Z, Hobson N, Galindo L, Zhu S, Shi D, McDill J, Yang L, Hawkins S, Neutelings G, Datla R *et al*: **The genome of flax (*Linum usitatissimum*) assembled *de novo* from short shotgun sequence reads**. *Plant J* 2012, **72**(3):461-473.

9. Velasco R, Zharkikh A, Affourtit J, Dhingra A, Cestaro A, Kalyanaraman A, Fontana P, Bhatnagar SK, Troggio M, Pruss D *et al*: **The genome of the domesticated apple (*Malus x domestica* Borkh.)**. *Nat Genet* 2010, **42**(10):833-839.

10. Prochnik S, Marri PR, Desany B, Rabinowicz PD, Kodira C, Mohiuddin M, Rodriguez F, Fauquet C, Tohme J, Harkins T *et al*: **The Cassava Genome: Current Progress, Future Directions**. *Trop Plant Biol* 2012, **5**(1):88-94.

11. Young ND, Debelle F, Oldroyd GE, Geurts R, Cannon SB, Udvardi MK, Benedito VA, Mayer KF, Gouzy J, Schoof H *et al*: **The Medicago genome provides insight into the evolution of rhizobial symbioses**. *Nature* 2011, **480**(7378):520-524.

12. Worden AZ, Lee JH, Mock T, Rouze P, Simmons MP, Aerts AL, Allen AE, Cuvelier ML, Derelle E, Everett MV *et al*: **Green evolution and dynamic adaptations revealed by genomes of the marine picoeukaryotes Micromonas**. *Science* 2009, **324**(5924):268-272.

13. Ouyang S, Zhu W, Hamilton J, Lin H, Campbell M, Childs K, Thibaud-Nissen F, Malek RL, Lee Y, Zheng L *et al*: **The TIGR Rice Genome Annotation Resource: improvements and new features**. *Nucleic Acids Res* 2007, **35**(Database issue):D883-887.

14. Palenik B, Grimwood J, Aerts A, Rouze P, Salamov A, Putnam N, Dupont C, Jorgensen R, Derelle E, Rombauts S *et al*: **The tiny eukaryote Ostreococcus provides genomic insights into the paradox of plankton speciation**. *Proc Natl Acad Sci U S A* 2007, **104**(18):7705-7710.

15. Rensing SA, Lang D, Zimmer AD, Terry A, Salamov A, Shapiro H, Nishiyama T, Perroud PF, Lindquist EA, Kamisugi Y *et al*: **The Physcomitrella genome reveals evolutionary insights into the conquest of land by plants**. *Science* 2008, **319**(5859):64-69.

16. Tuskan GA, Difazio S, Jansson S, Bohlmann J, Grigoriev I, Hellsten U, Putnam N, Ralph S, Rombauts S, Salamov A *et al*: **The genome of black cottonwood, *Populus trichocarpa* (Torr. & Gray)**. *Science* 2006, **313**(5793):1596-1604.

17. Chan AP, Crabtree J, Zhao Q, Lorenzi H, Orvis J, Puiu D, Melake-Berhan A, Jones KM, Redman J, Chen G *et al*: **Draft genome sequence of the oilseed species *Ricinus communis***. *Nat Biotechnol* 2010, **28**(9):951-956.

18. Banks JA, Nishiyama T, Hasebe M, Bowman JL, Gribskov M, dePamphilis C, Albert VA, Aono N, Aoyama T, Ambrose BA *et al*: **The Selaginella genome identifies genetic changes associated with the evolution of vascular plants**. *Science* 2011, **332**(6032):960-963.

19. Bennetzen JL, Schmutz J, Wang H, Percifield R, Hawkins J, Pontaroli AC, Estep M, Feng L, Vaughn JN, Grimwood J *et al*: **Reference genome sequence of the model plant Setaria**. *Nat Biotechnol* 2012, **30**(6):555-561.

20. **The tomato genome sequence provides insights into fleshy fruit evolution**. *Nature* 2012, **485**(7400):635-641.

21. Xu X, Pan S, Cheng S, Zhang B, Mu D, Ni P, Zhang G, Yang S, Li R, Wang J *et al*: **Genome sequence and analysis of the tuber crop potato**. *Nature* 2011, **475**(7355):189-195.

22. Paterson AH, Bowers JE, Bruggmann R, Dubchak I, Grimwood J, Gundlach H, Haberer G, Hellsten U, Mitros T, Poliakov A *et al*: **The *Sorghum bicolor* genome and the diversification of grasses**. *Nature* 2009, **457**(7229):551-556.

23. Jaillon O, Aury JM, Noel B, Policriti A, Clepet C, Casagrande A, Choisne N, Aubourg S, Vitulo N, Jubin C *et al*: **The grapevine genome sequence suggests ancestral hexaploidization in major angiosperm phyla**. *Nature* 2007, **449**(7161):463-467.

24. Prochnik SE, Umen J, Nedelcu AM, Hallmann A, Miller SM, Nishii I, Ferris P, Kuo A, Mitros T, Fritz-Laylin LK *et al*: **Genomic analysis of organismal complexity in the multicellular green alga Volvox carteri**. *Science* 2010, **329**(5988):223-226.

25. Schnable PS, Ware D, Fulton RS, Stein JC, Wei F, Pasternak S, Liang C, Zhang J, Fulton L, Graves TA *et al*: **The B73 maize genome: complexity, diversity, and dynamics**. *Science* 2009, **326**(5956):1112-1115.
