# Supplementary material for: Codon usage patterns in Chinese bayberry (Myrica rubra) based on RNA-Seq data
Source: BMC Genomics. 2013 Oct 25;14:732. doi: 10.1186/1471-2164-14-732 (PMC4008310; doi:10.1186/1471-2164-14-732)
Supplement: Additional file 4 — 18 groups of high-frequency codon pairs for codons for 18 amino acids (expecting Met and Trp) in Chinese bayberry. 64 codons were put in a clockwise order of 'U’, 'C’, 'A’ and 'G’. Codons encoding the same amino acid or stop codons, as well as the amino acid, were indicated with the same color. The arrow lines represent high-frequency codon pairs; the direction of the arrow links the first codon to the one following, and the color is the same as that of the following codons. [file 1471-2164-14-732-S4.pdf]

Additional file 4. 18 groups of high-frequency codon pairs for codons for 18 amino acids (expecting Met and Trp) in Chinese bayberry. 64 codons were put in a clockwise order of 'U', 'C', 'A' and 'G'. Codons encoding the same amino acid or stop codons, as well as the amino acid, were indicated with the same color. The arrow lines represent high-frequency codon pairs; the direction of the arrow links the first codon to the one following, and the color is the same as that of the following codons.

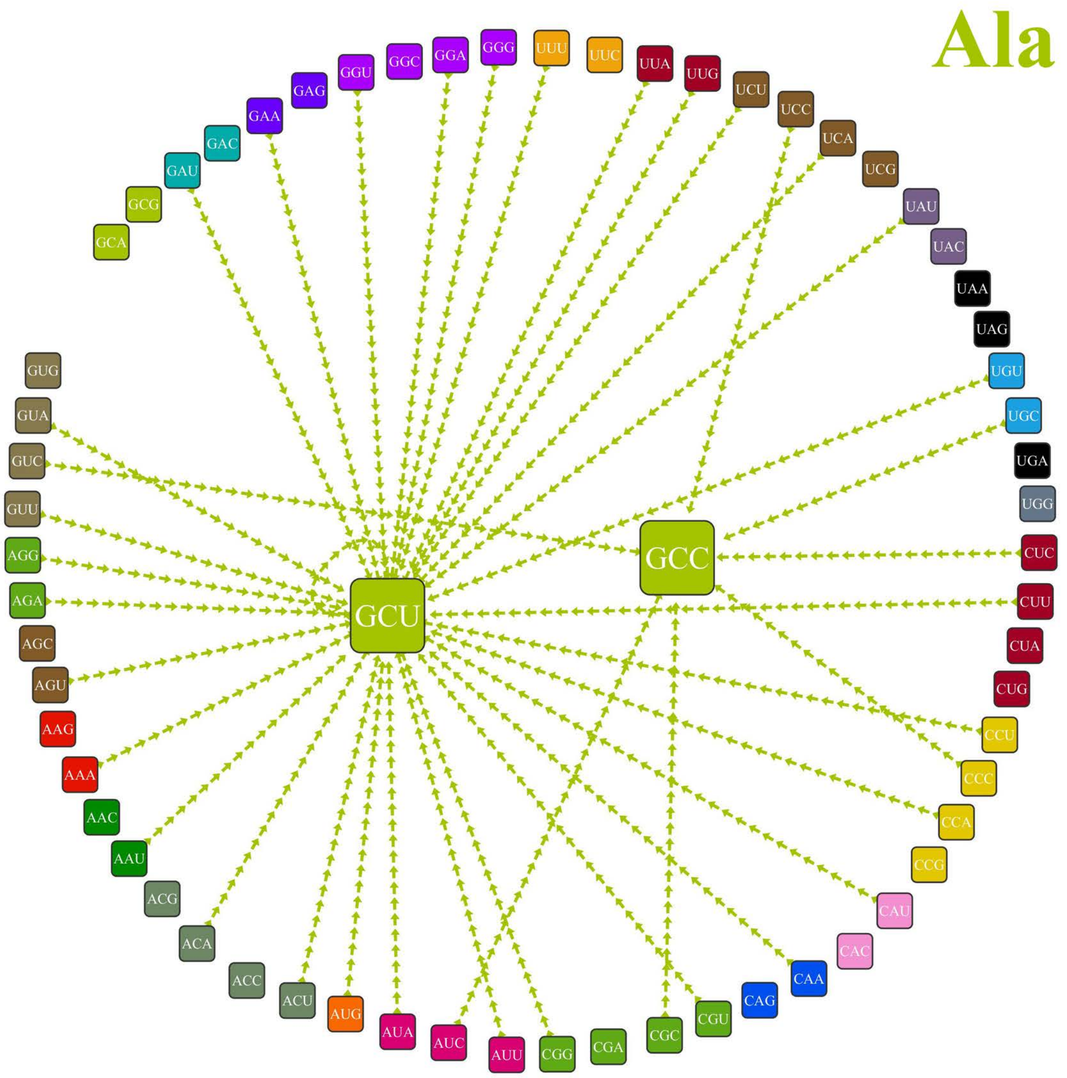

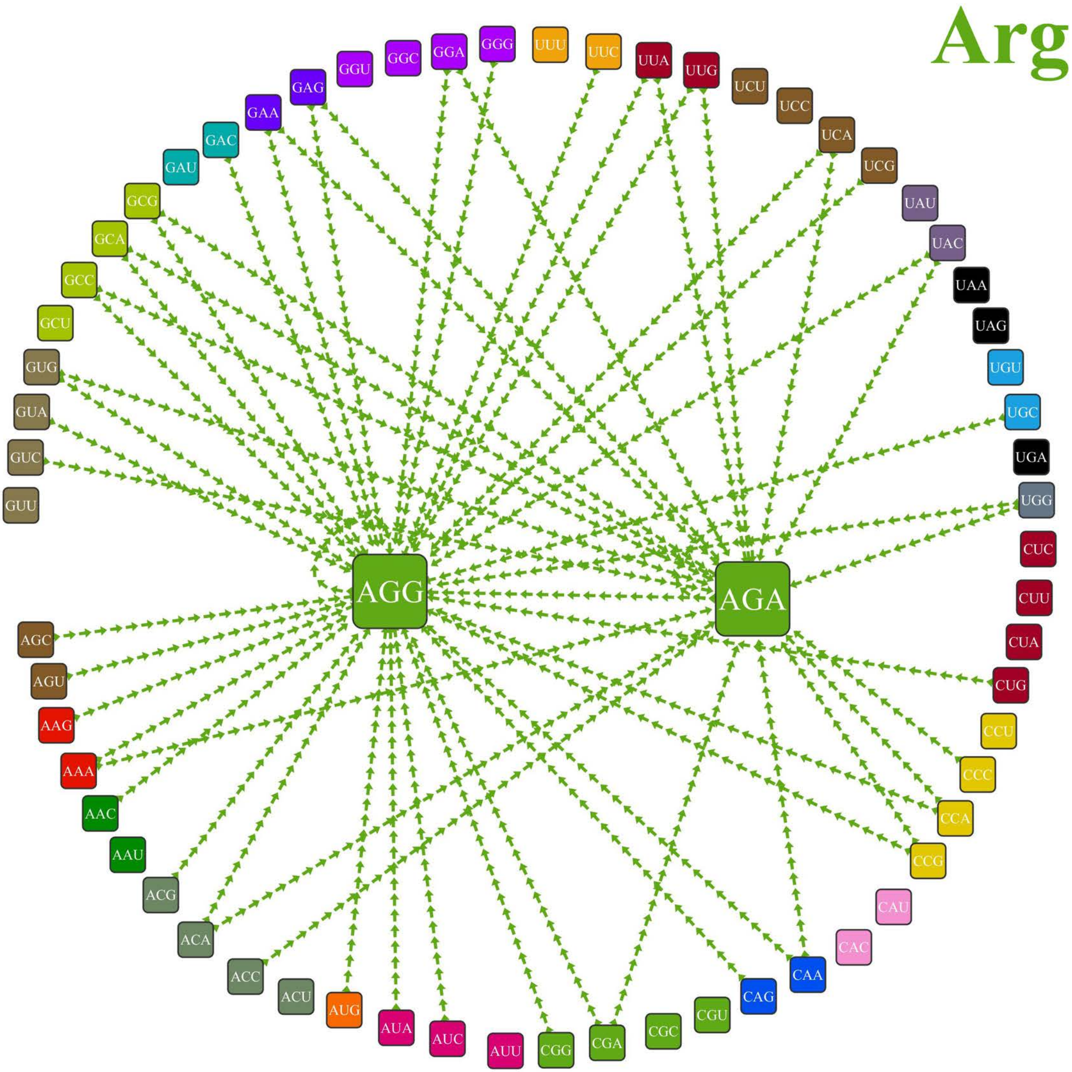
Arg
AGG
AGA
GGG
GGA
GGC
GGU
GAG
GAA
GAC
GAU
GCG
GCA
GCC
GCU
GUG
GUA
GUC
GUU
UUU
UUC
UUA
UUG
UCU
UCC
UCA
UCG
UAU
UAC
UAA
UAG
UGU
UGC
UGA
UGG
CUC
CUU
CUA
CUG
CCU
CCC
CCA
CCG
CAU
CAC
CAA
CAG
CGU
CGC
CGA
CGG
AUU
AUC
AUA
AUG
ACU
ACC
ACA
ACG
AAU
AAC
AAA
AAG
AGU
AGC

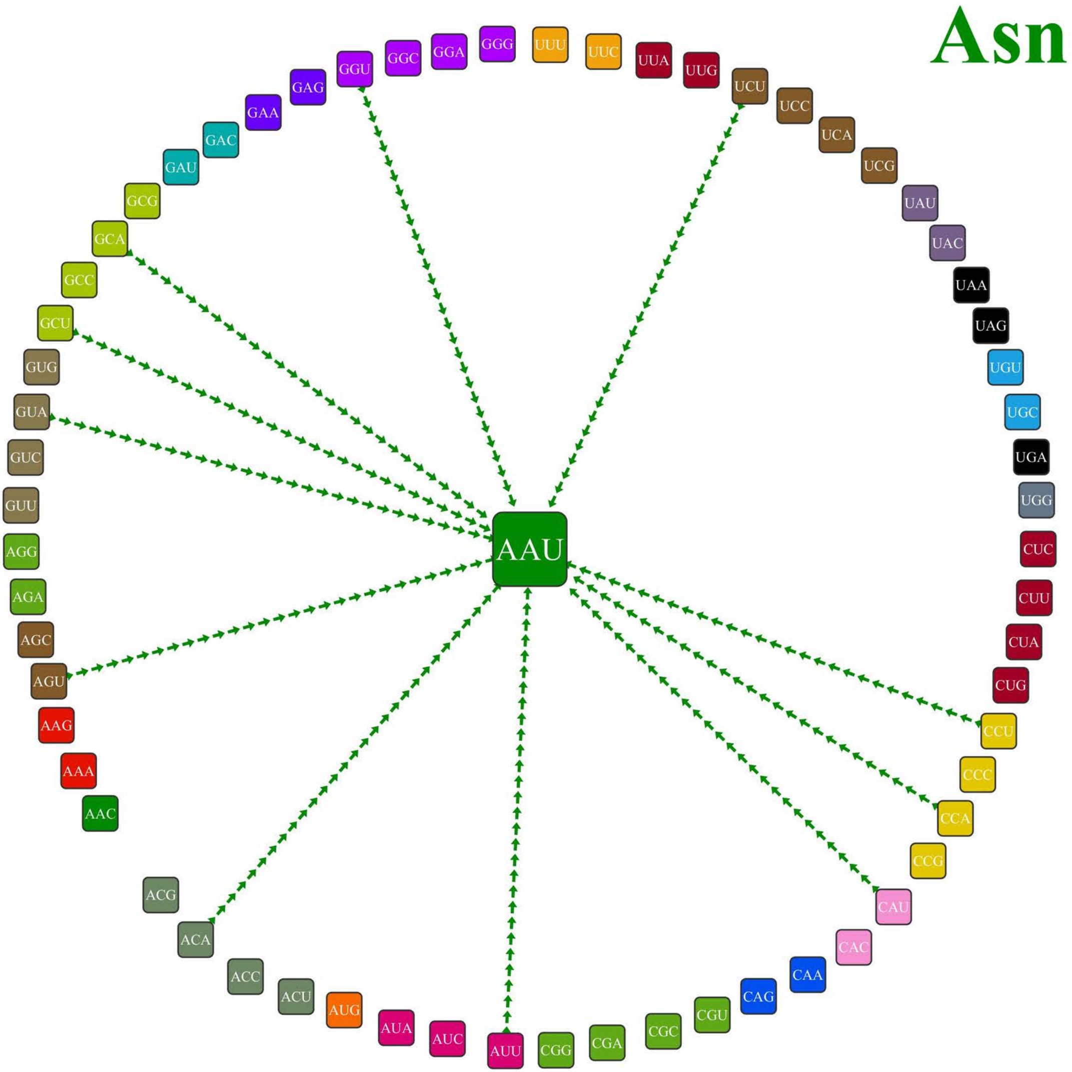
Asn
AAU
GGU
GGC
GGA
GGG
UUU
UUC
UUA
UUG
UCU
UCC
UCA
UCG
UAU
UAC
UAA
UAG
UGU
UGC
UGA
UGG
CUC
CUU
CUA
CUG
CCU
CCC
CCA
CCG
CAU
CAC
CAA
CAG
CGU
CGC
CGA
CGG
AUU
AUC
AUA
AUG
ACU
ACC
ACA
ACG
AAC
AAA
AAG
AGU
AGC
AGA
AGG
GUU
GUC
GUA
GUG
GCU
GCC
GCA
GCG
GAU
GAC
GAA
GAG

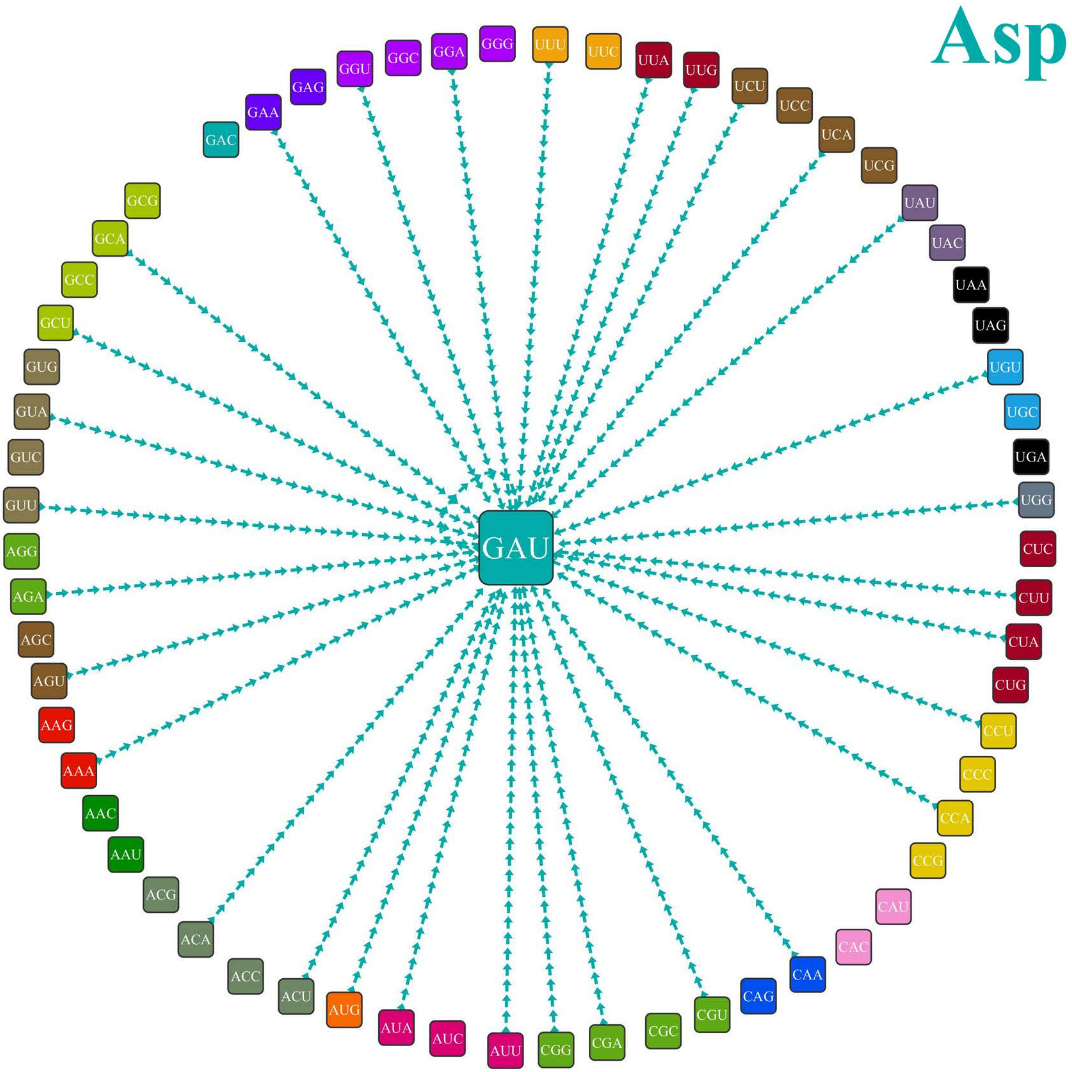
Asp
GAU
GAC
GAA
GAG
GGU
GGC
GGA
GGG
UUU
UUC
UUA
UUG
UCU
UCC
UCA
UCG
UAU
UAC
UAA
UAG
UGU
UGC
UGA
UGG
CUC
CUU
CUA
CUG
CCU
CCC
CCA
CCG
CAU
CAC
CAA
CAG
CGU
CGC
CGA
CGG
AUU
AUC
AUA
AUG
ACU
ACC
ACA
ACG
AAU
AAC
AAA
AAG
AGU
AGC
AGA
AGG
GUU
GUC
GUA
GUG
GCU
GCC
GCA
GCG

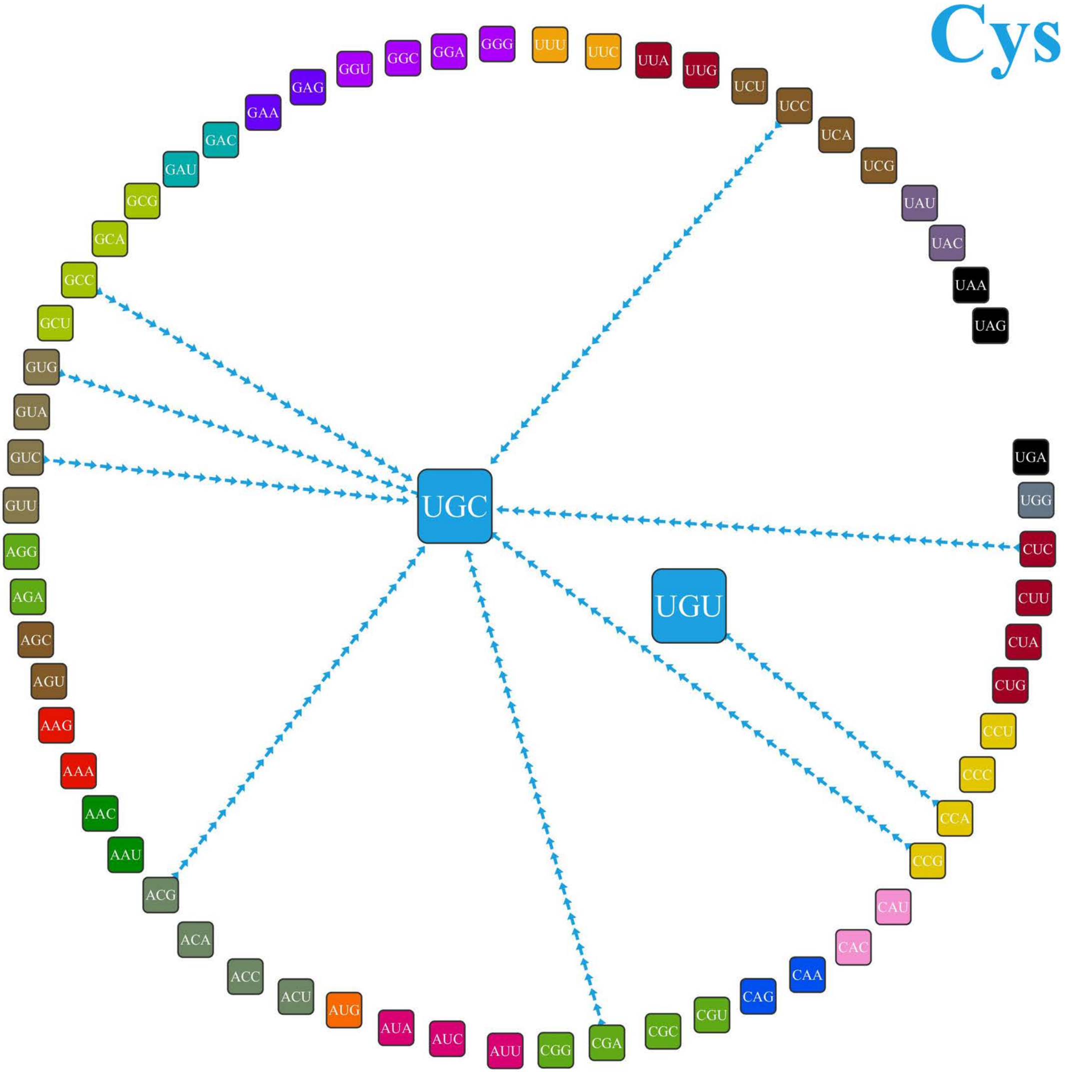
Cys
UGC
UGU
GGG
UUU
UUC
UUA
UUG
UCU
UCC
UCA
UCG
UAU
UAC
UAA
UAG
UGA
UGG
CUC
CUU
CUA
CUG
CCU
CCC
CCA
CCG
CAU
CAC
CAA
CAG
CGU
CGC
CGA
CGG
AUU
AUC
AUA
AUG
ACU
ACC
ACA
ACG
AAU
AAC
AAA
AAG
AGU
AGC
AGA
AGG
GUU
GUC
GUA
GUG
GCU
GCC
GCA
GCG
GAU
GAC
GAA
GAG
GGU
GGC
GGA

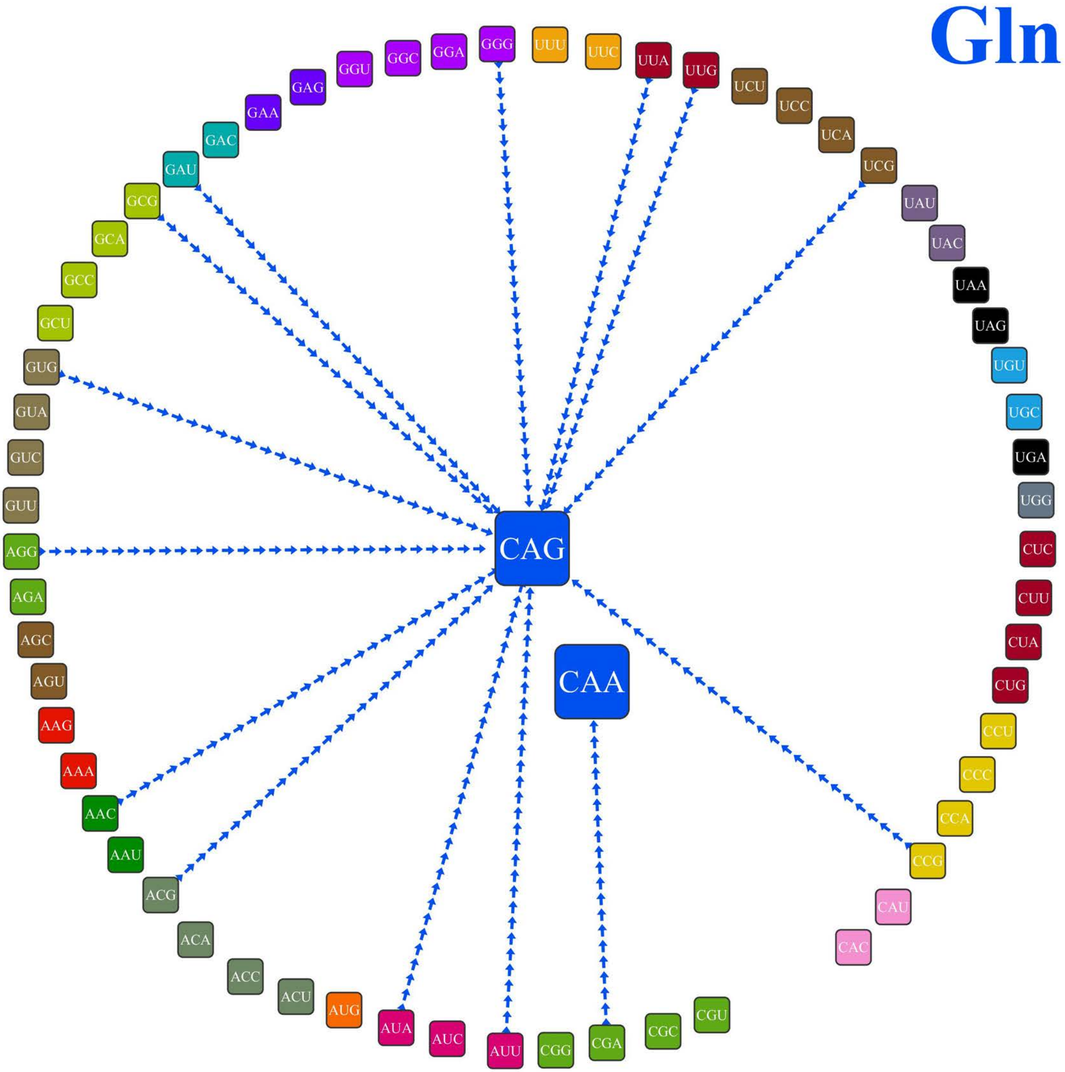

Gln
CAG
CAA
GGG
UUU
UUC
UUA
UUG
UCU
UCC
UCA
UCG
UAU
UAC
UAA
UAG
UGU
UGC
UGA
UGG
CUC
CUU
CUA
CUG
CCU
CCC
CCA
CCG
CAU
CAC
CGU
CGC
CGA
CGG
AUU
AUC
AUA
AUG
ACU
ACC
ACA
ACG
AAU
AAC
AAA
AAG
AGU
AGC
AGA
AGG
GUU
GUC
GUA
GUG
GCU
GCC
GCA
GCG
GAU
GAC
GAA
GAG
GGU
GGC
GGA

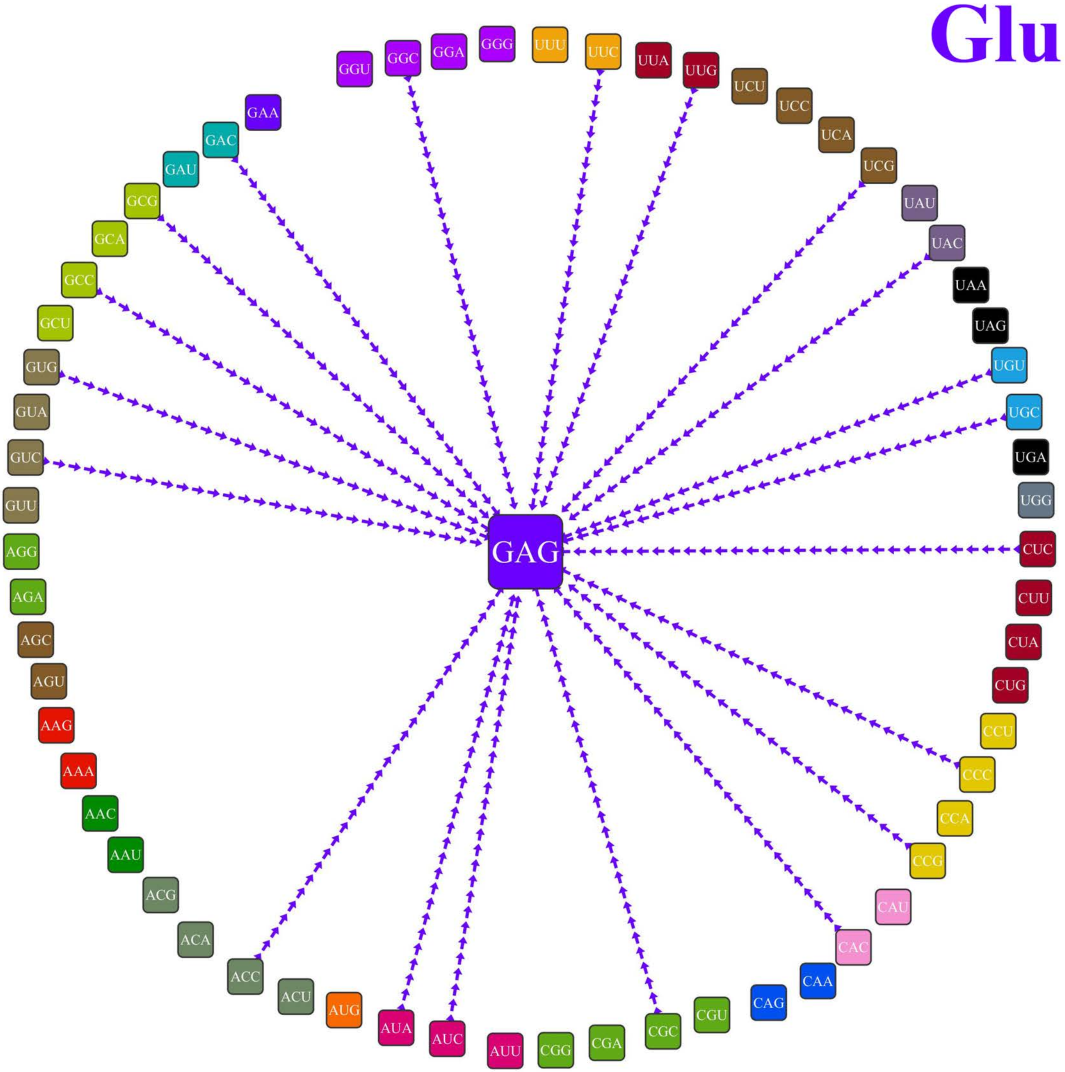
Glu
GAG
GGU
GGC
GGA
GGG
UUU
UUC
UUA
UUG
UCU
UCC
UCA
UCG
UAU
UAC
UAA
UAG
UGU
UGC
UGA
UGG
CUC
CUU
CUA
CUG
CCU
CCC
CCA
CCG
CAU
CAC
CAA
CAG
CGU
CGC
CGA
CGG
AUU
AUC
AUA
AUG
ACU
ACC
ACA
ACG
AAU
AAC
AAA
AAG
AGU
AGC
AGA
AGG
GUU
GUC
GUA
GUG
GCU
GCC
GCA
GCG
GAU
GAC
GAA

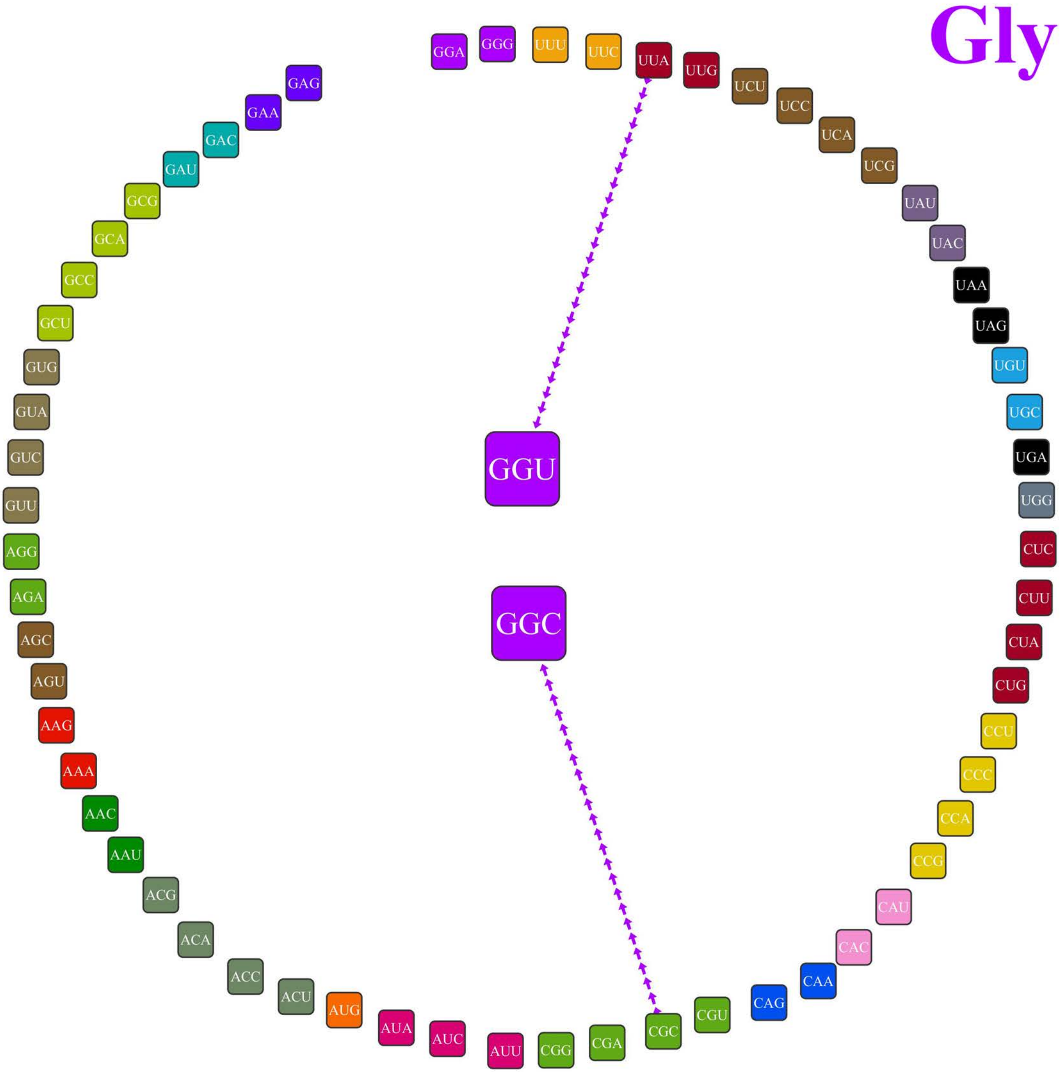

Gly
GGA
GGG
UUU
UUC
UUA
UUG
UCU
UCC
UCA
UCG
UAU
UAC
UAA
UAG
UGU
UGC
UGA
UGG
CUC
CUU
CUA
CUG
CCU
CCC
CCA
CCG
CAU
CAC
CAA
CAG
CGU
CGC
CGA
CGG
AUU
AUC
AUA
AUG
ACU
ACC
ACA
ACG
AAU
AAC
AAA
AAG
AGU
AGC
AGA
AGG
GUU
GUC
GUA
GUG
GCU
GCC
GCA
GCG
GAU
GAC
GAA
GAG
GGU
GGC

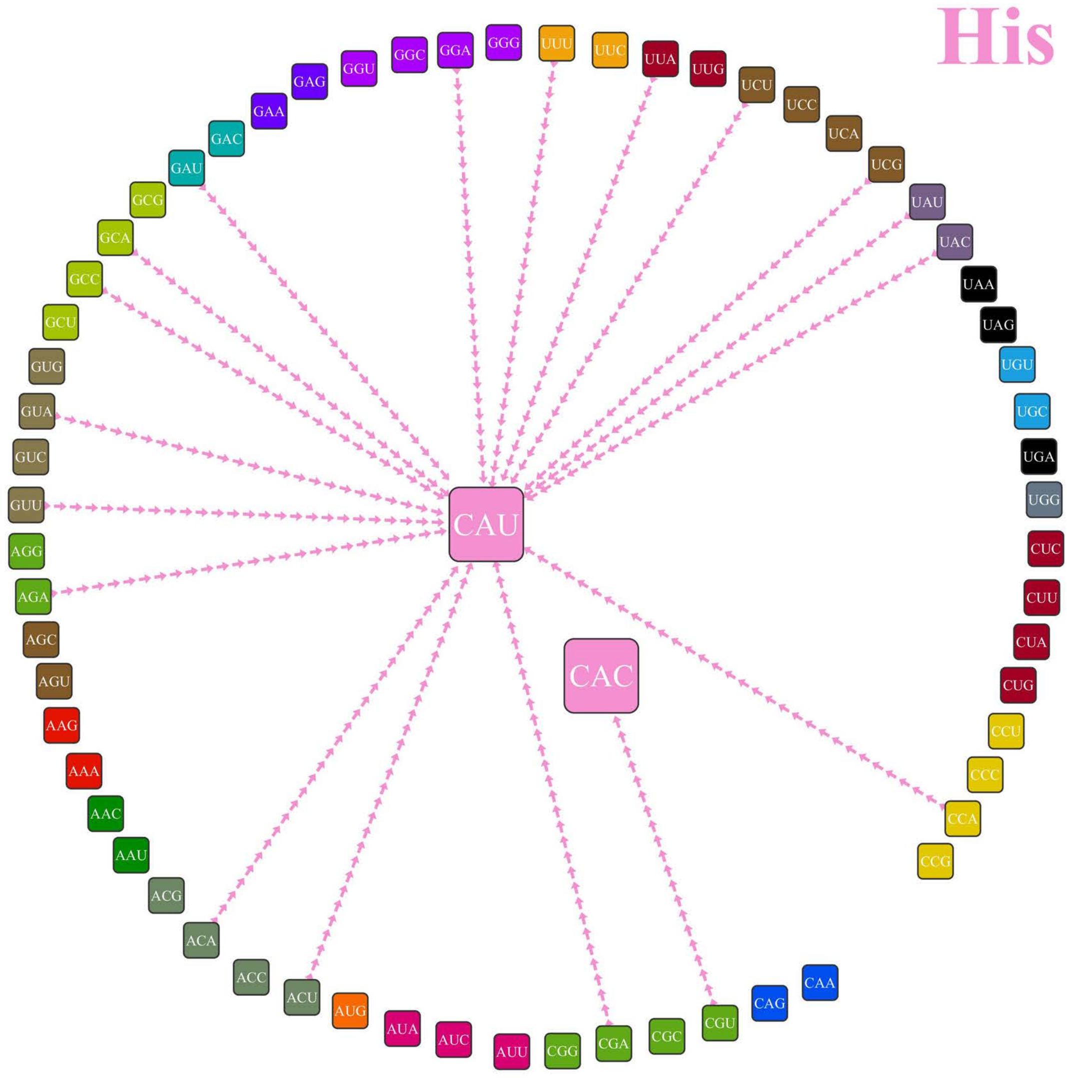
His
CAU
CAC
GGA
GGG
UUU
UUC
UUA
UUG
UCU
UCC
UCA
UCG
UAU
UAC
UAA
UAG
UGU
UGC
UGA
UGG
CUC
CUU
CUA
CUG
CCU
CCC
CCA
CCG
CAA
CAG
CGU
CGC
CGA
CGG
AUU
AUC
AUA
AUG
ACU
ACC
ACA
ACG
AAU
AAC
AAA
AAG
AGU
AGC
AGA
AGG
GUU
GUC
GUA
GUG
GCU
GCC
GCA
GCG
GAU
GAC
GAA
GAG
GGU
GGC

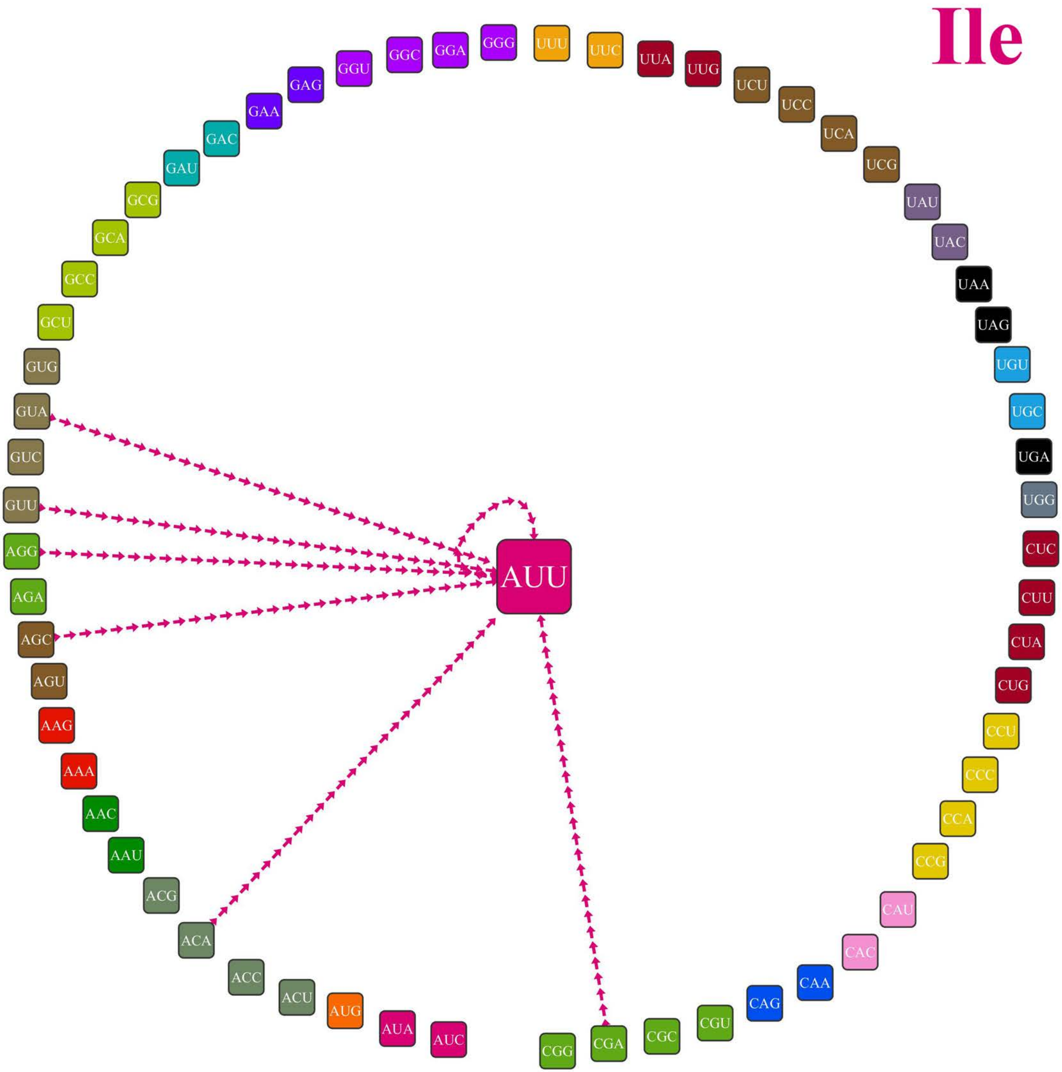
Ile
AUU
UUU
UUC
UUA
UUG
UCU
UCC
UCA
UCG
UAU
UAC
UAA
UAG
UGU
UGC
UGA
UGG
CUC
CUU
CUA
CUG
CCU
CCC
CCA
CCG
CAU
CAC
CAA
CAG
CGU
CGC
CGA
CGG
AUC
AUA
AUG
ACU
ACC
ACA
ACG
AAU
AAC
AAA
AAG
AGU
AGC
AGA
AGG
GUU
GUC
GUA
GUG
GCU
GCC
GCA
GCG
GAU
GAC
GAA
GAG
GGU
GGC
GGA
GGG

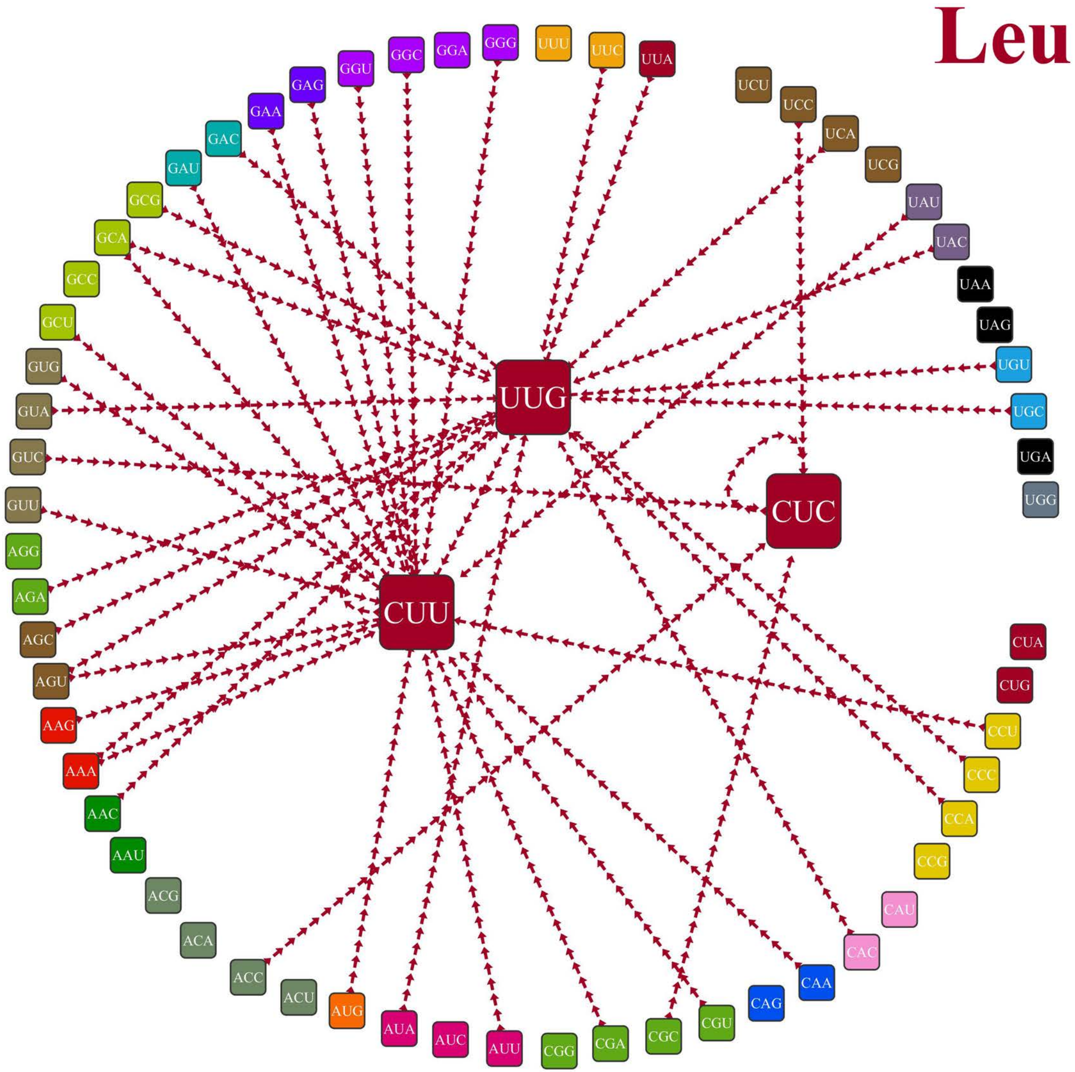
Leu
UUG
CUU
CUC
GGG
UUU
UUC
UUA
GGA
GGC
GGU
GAG
GAA
GAC
GAU
GCG
GCA
GCC
GCU
GUG
GUA
GUC
GUU
AGG
AGA
AGC
AGU
AAG
AAA
AAC
AAU
ACG
ACA
ACC
ACU
AUG
AUA
AUC
AUU
CGG
CGA
CGC
CGU
CAG
CAA
CAC
CAU
CCG
CCA
CCC
CCU
CUG
CUA
UCU
UCC
UCA
UCG
UAU
UAC
UAA
UAG
UGU
UGC
UGA
UGG

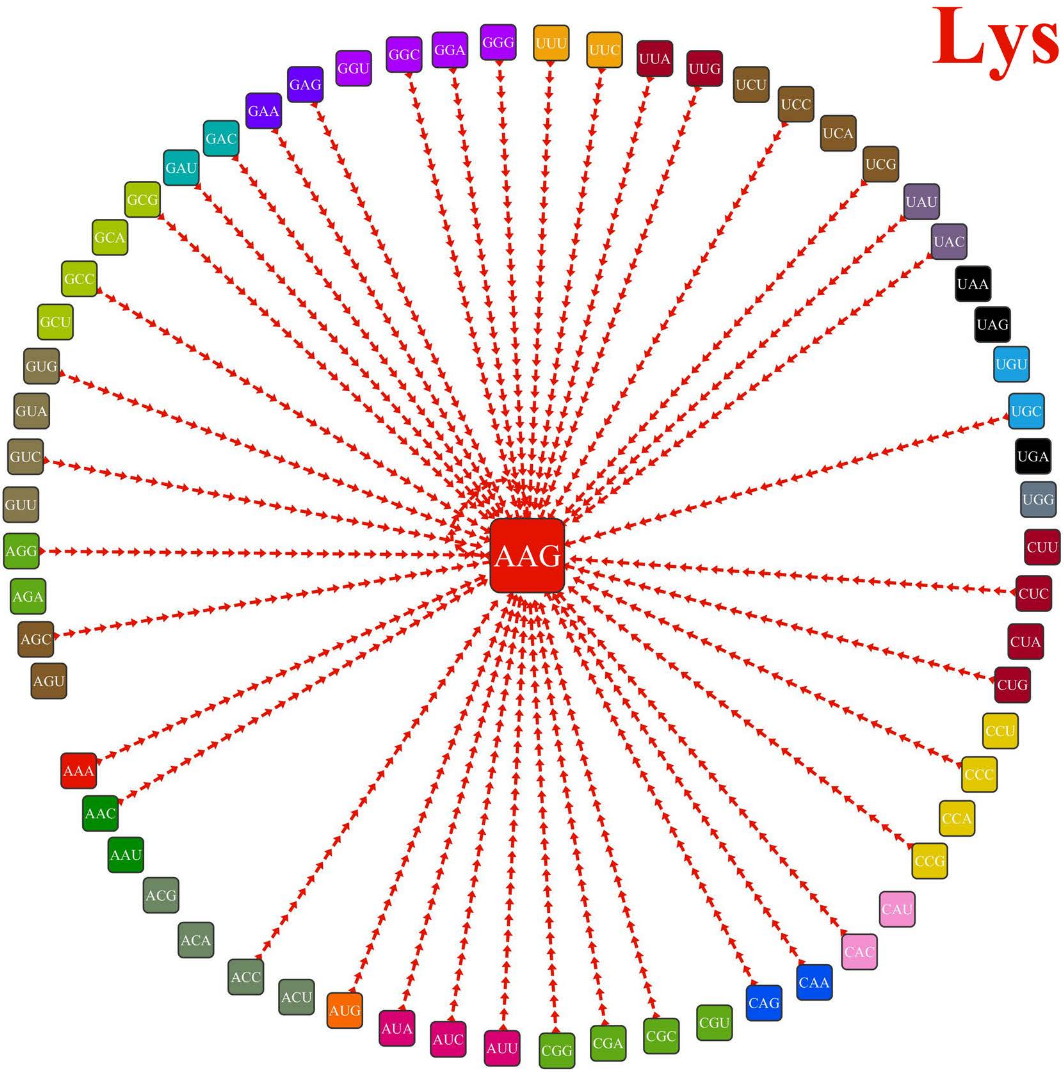

Lys
AAG
GGG
UUU
UUC
UUA
UUG
UCU
UCC
UCA
UCG
UAU
UAC
UAA
UAG
UGU
UGC
UGA
UGG
CUU
CUC
CUA
CUG
CCU
CCC
CCA
CCG
CAU
CAC
CAA
CAG
CGU
CGC
CGA
CGG
AUU
AUC
AUA
AUG
ACU
ACC
ACA
ACG
AAU
AAC
AAA
AGU
AGC
AGA
AGG
GUU
GUC
GUA
GUG
GCU
GCC
GCA
GCG
GAU
GAC
GAA
GAG
GGU
GGC
GGA

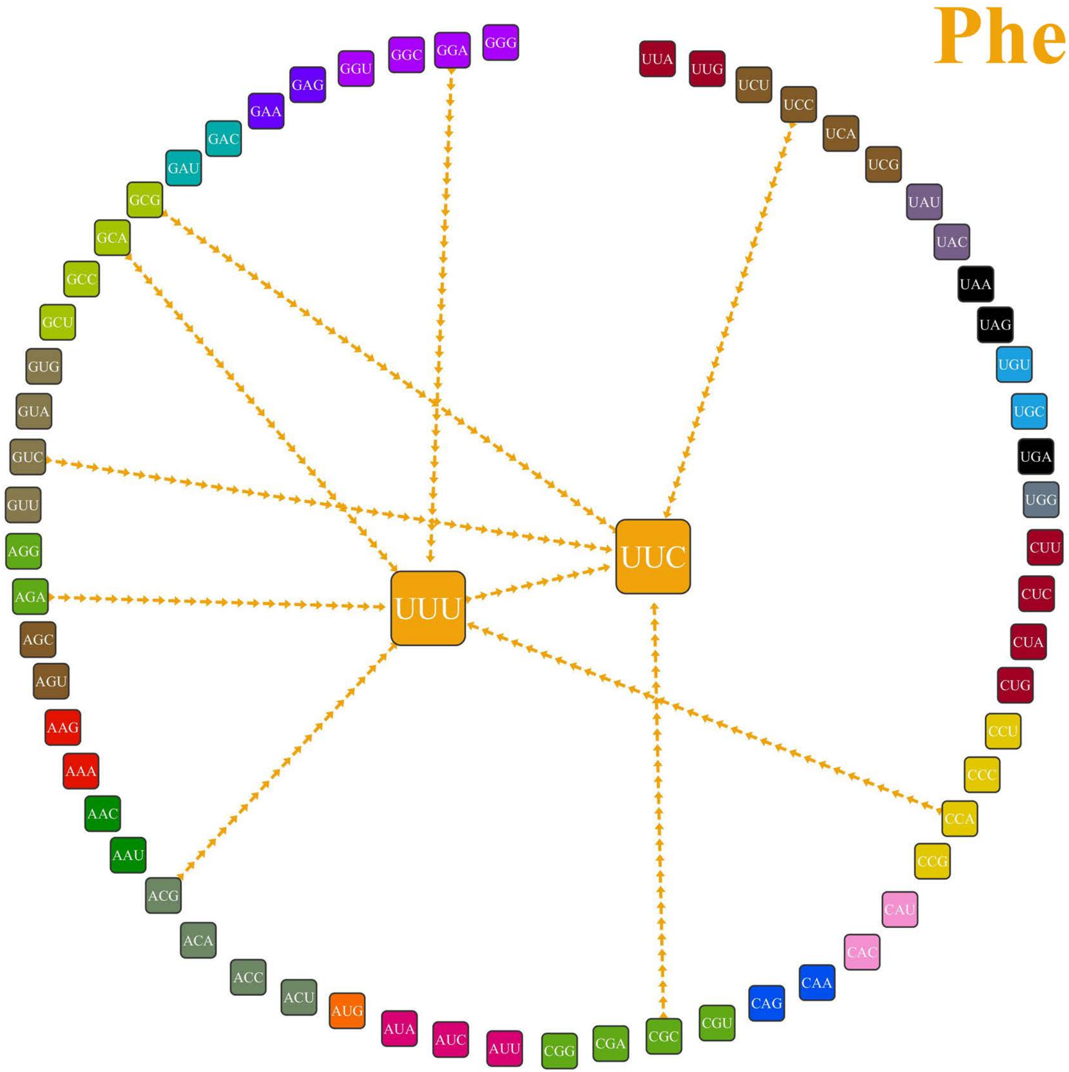
Phe
UUU
UUC
UUA
UUG
UCU
UCC
UCA
UCG
UAU
UAC
UAA
UAG
UGU
UGC
UGA
UGG
CUU
CUC
CUA
CUG
CCU
CCC
CCA
CCG
CAU
CAC
CAA
CAG
CGU
CGC
CGA
CGG
AUU
AUC
AUA
AUG
ACU
ACC
ACA
ACG
AAU
AAC
AAA
AAG
AGU
AGC
AGA
AGG
GUU
GUC
GUA
GUG
GCU
GCC
GCA
GCG
GAU
GAC
GAA
GAG
GGU
GGC
GGA
GGG

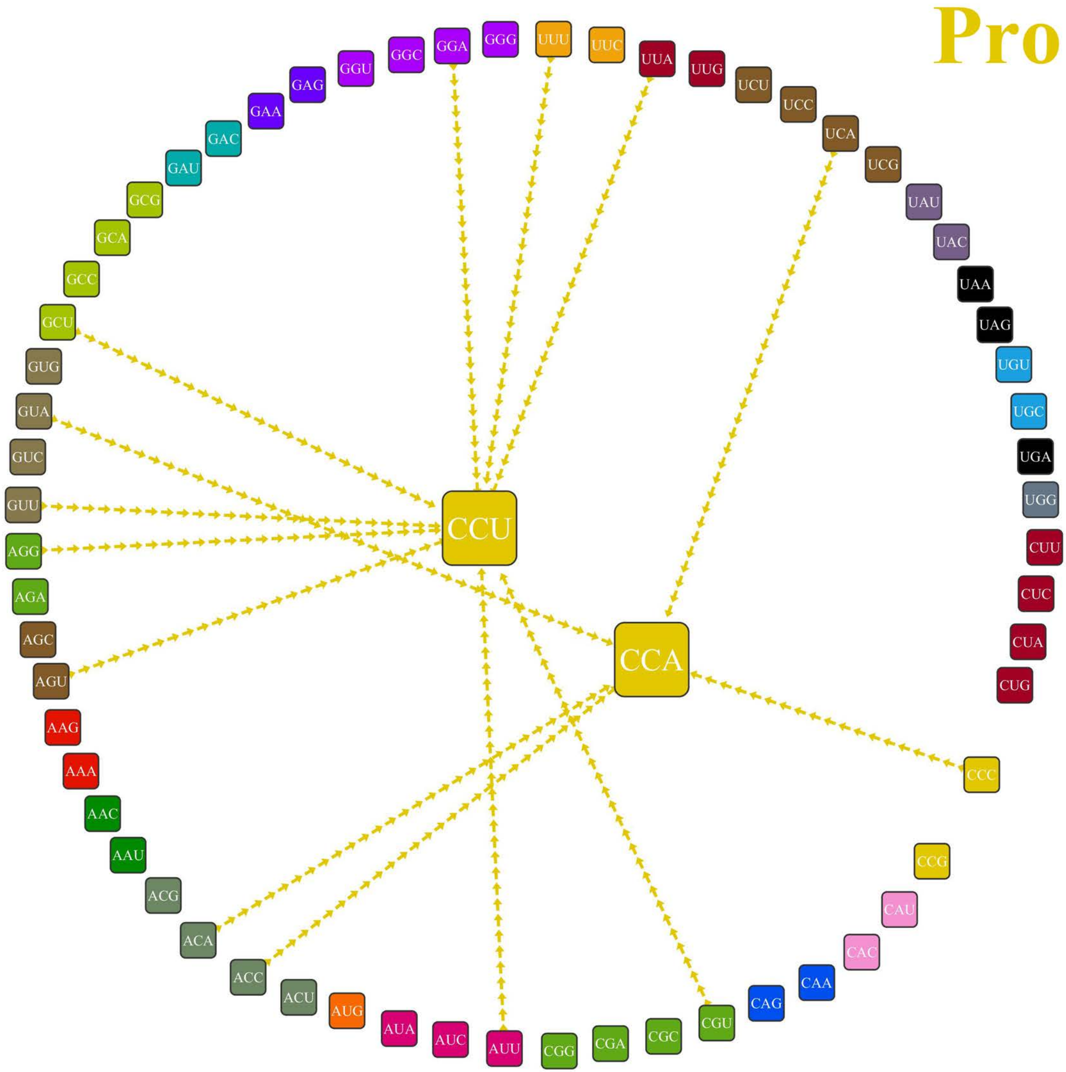

Pro
CCU
CCA
GGA
GGG
UUU
UUC
UUA
UUG
UCU
UCC
UCA
UCG
UAU
UAC
UAA
UAG
UGU
UGC
UGA
UGG
CUU
CUC
CUA
CUG
CCC
CCG
CAU
CAC
CAA
CAG
CGU
CGC
CGA
CGG
AUU
AUC
AUA
AUG
ACU
ACC
ACA
ACG
AAU
AAC
AAA
AAG
AGU
AGC
AGA
AGG
GUU
GUC
GUA
GUG
GCU
GCC
GCA
GCG
GAU
GAC
GAA
GAG
GGU
GGC

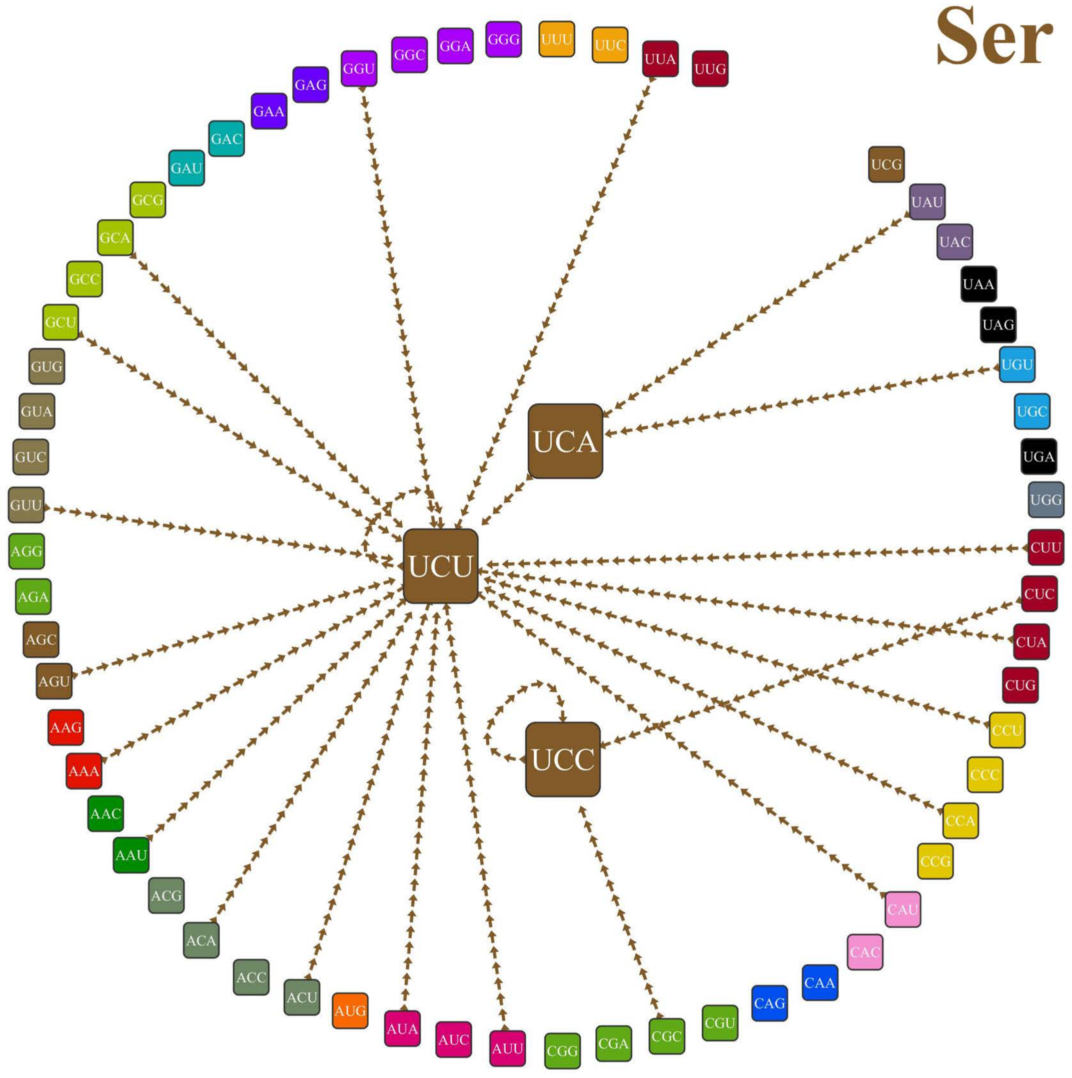
Ser
UCU
UCA
UCC
GGU
GGC
GGA
GGG
UUU
UUC
UUA
UUG
GAG
GAA
GAC
GAU
GCG
GCA
GCC
GCU
GUG
GUA
GUC
GUU
AGG
AGA
AGC
AGU
AAG
AAA
AAC
AAU
ACG
ACA
ACC
ACU
AUG
AUA
AUC
AUU
CGG
CGA
CGC
CGU
CAG
CAA
CAC
CAU
CCG
CCA
CCC
CCU
CUG
CUA
CUC
CUU
UGG
UGA
UGC
UGU
UAG
UAA
UAC
UAU
UCG

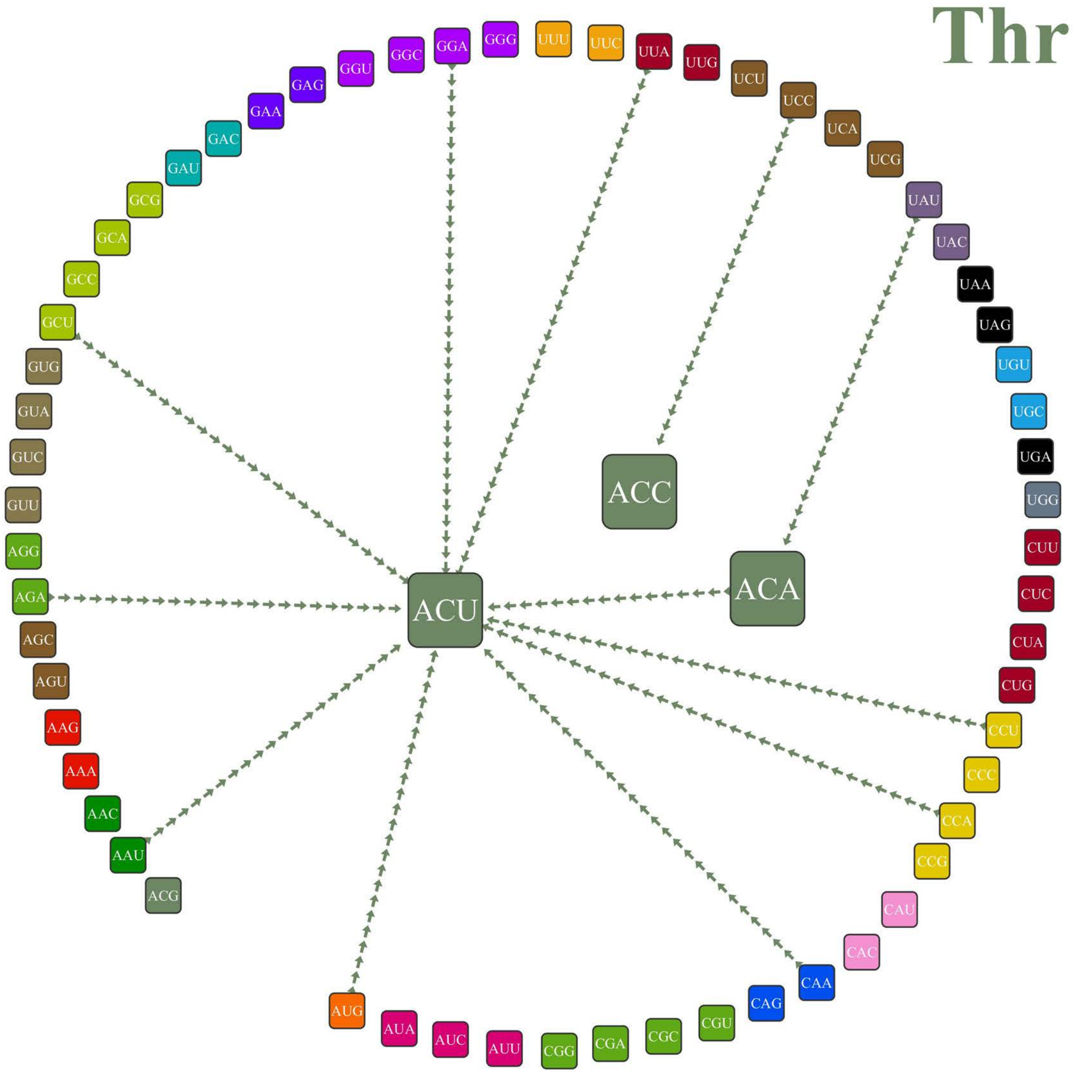

Thr
ACU
ACC
ACA
GGG
UUU
UUC
UUA
UUG
UCU
UCC
UCA
UCG
UAU
UAC
UAA
UAG
UGU
UGC
UGA
UGG
CUU
CUC
CUA
CUG
CCU
CCC
CCA
CCG
CAU
CAC
CAA
CAG
CGU
CGC
CGA
CGG
AUU
AUC
AUA
AUG
ACG
AAU
AAC
AAA
AAG
AGU
AGC
AGA
AGG
GUU
GUC
GUA
GUG
GCU
GCC
GCA
GCG
GAU
GAC
GAA
GAG
GGU
GGC
GGA

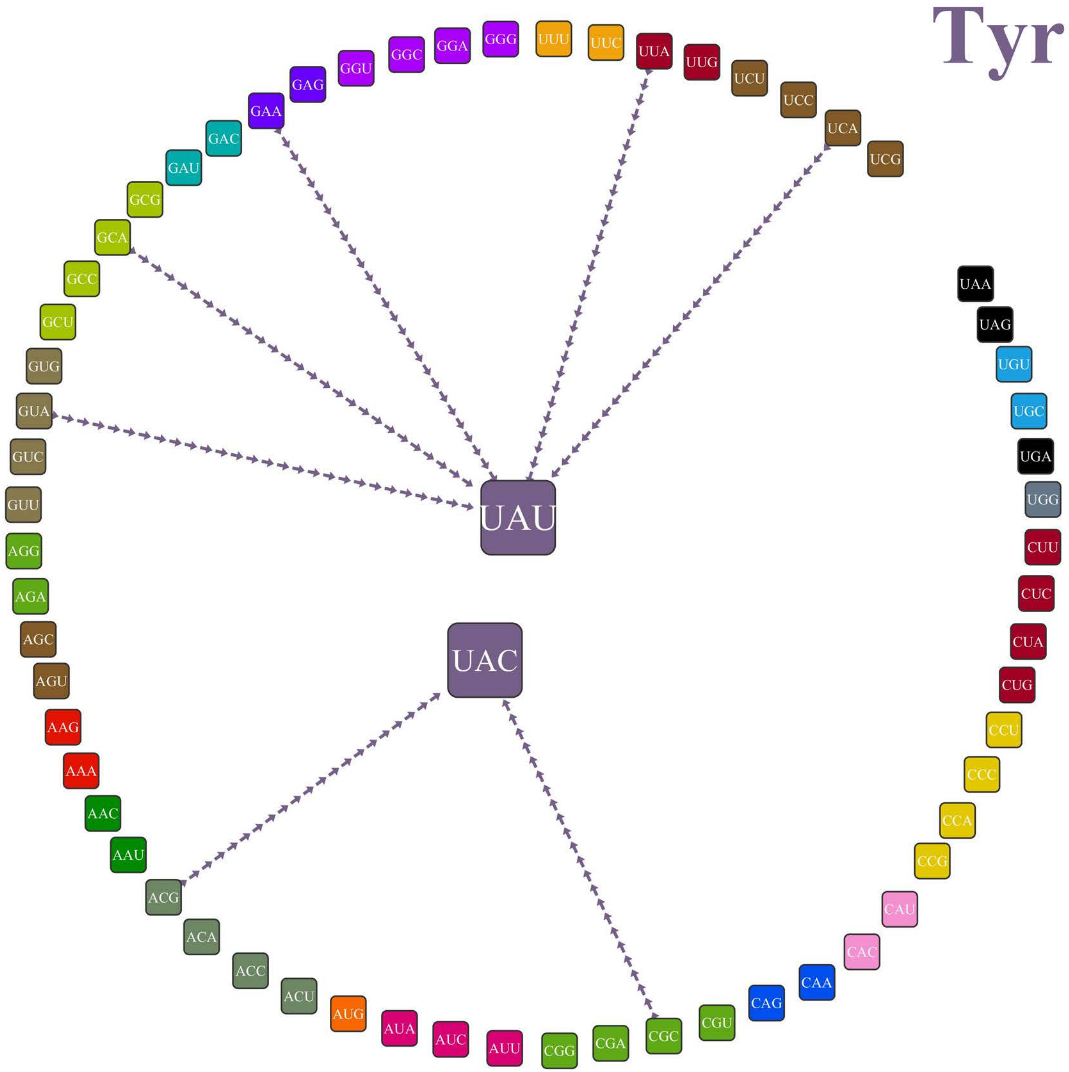
Tyr
UAU
UAC
UUU
UUC
UUA
UUG
UCU
UCC
UCA
UCG
UAA
UAG
UGU
UGC
UGA
UGG
CUU
CUC
CUA
CUG
CCU
CCC
CCA
CCG
CAU
CAC
CAA
CAG
CGU
CGC
CGA
CGG
AUU
AUC
AUA
AUG
ACU
ACC
ACA
ACG
AAU
AAC
AAA
AAG
AGU
AGC
AGA
AGG
GUU
GUC
GUA
GUG
GCU
GCC
GCA
GCG
GAU
GAC
GAA
GAG
GGU
GGC
GGA
GGG

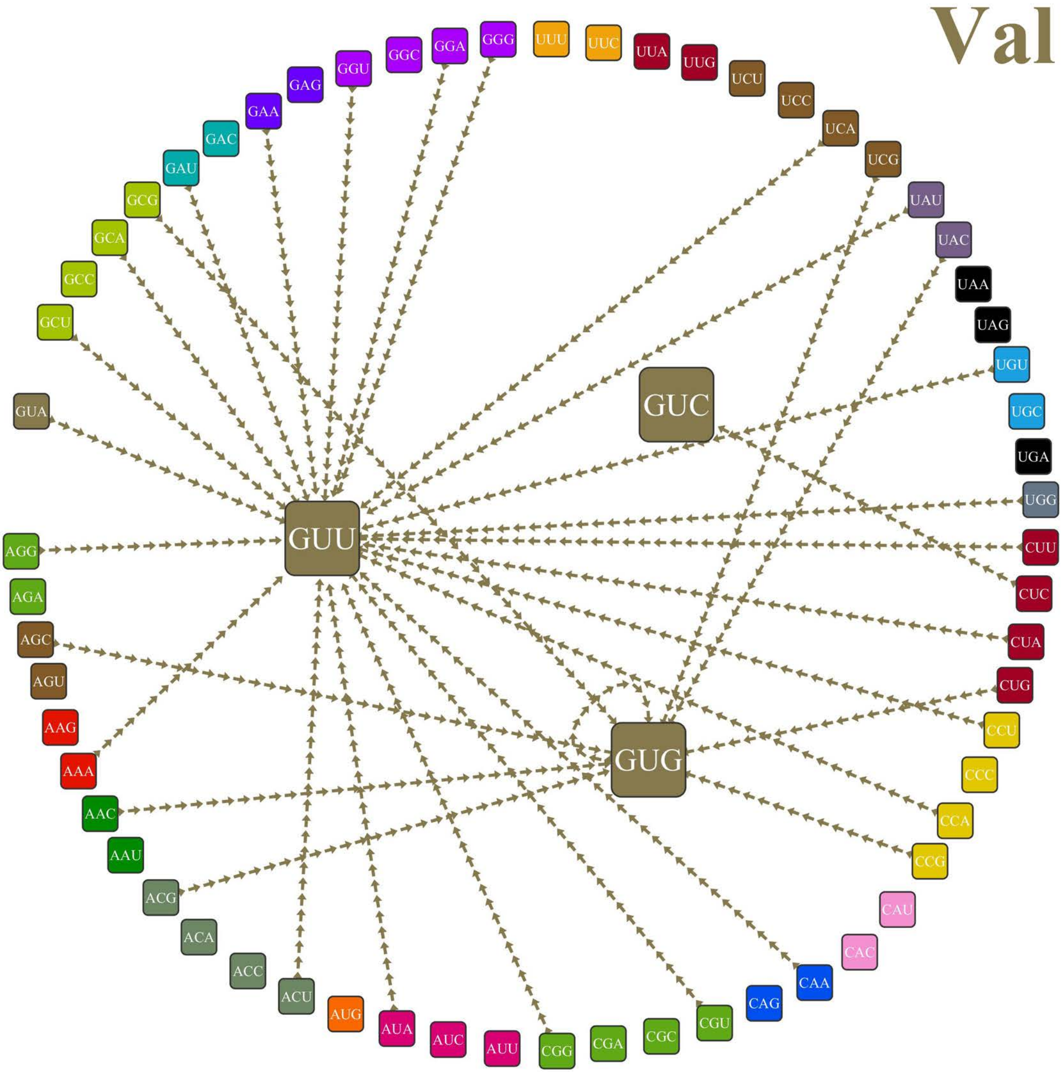
Val
GUU
GUC
GUG
GUA
GCU
GCC
GCA
GCG
GAU
GAC
GAA
GAG
GGU
GGC
GGA
GGG
UUU
UUC
UUA
UUG
UCU
UCC
UCA
UCG
UAU
UAC
UAA
UAG
UGU
UGC
UGA
UGG
CUU
CUC
CUA
CUG
CCU
CCC
CCA
CCG
CAU
CAC
CAA
CAG
CGU
CGC
CGA
CGG
AUU
AUC
AUA
AUG
ACU
ACC
ACA
ACG
AAU
AAC
AAA
AAG
AGU
AGC
AGA
AGG
