# Supplementary figures and images for: Codon usage patterns in Chinese bayberry (Myrica rubra) based on RNA-Seq data
Source: BMC Genomics. 2013 Oct 25;14:732. doi: 10.1186/1471-2164-14-732 (PMC4008310; doi:10.1186/1471-2164-14-732)

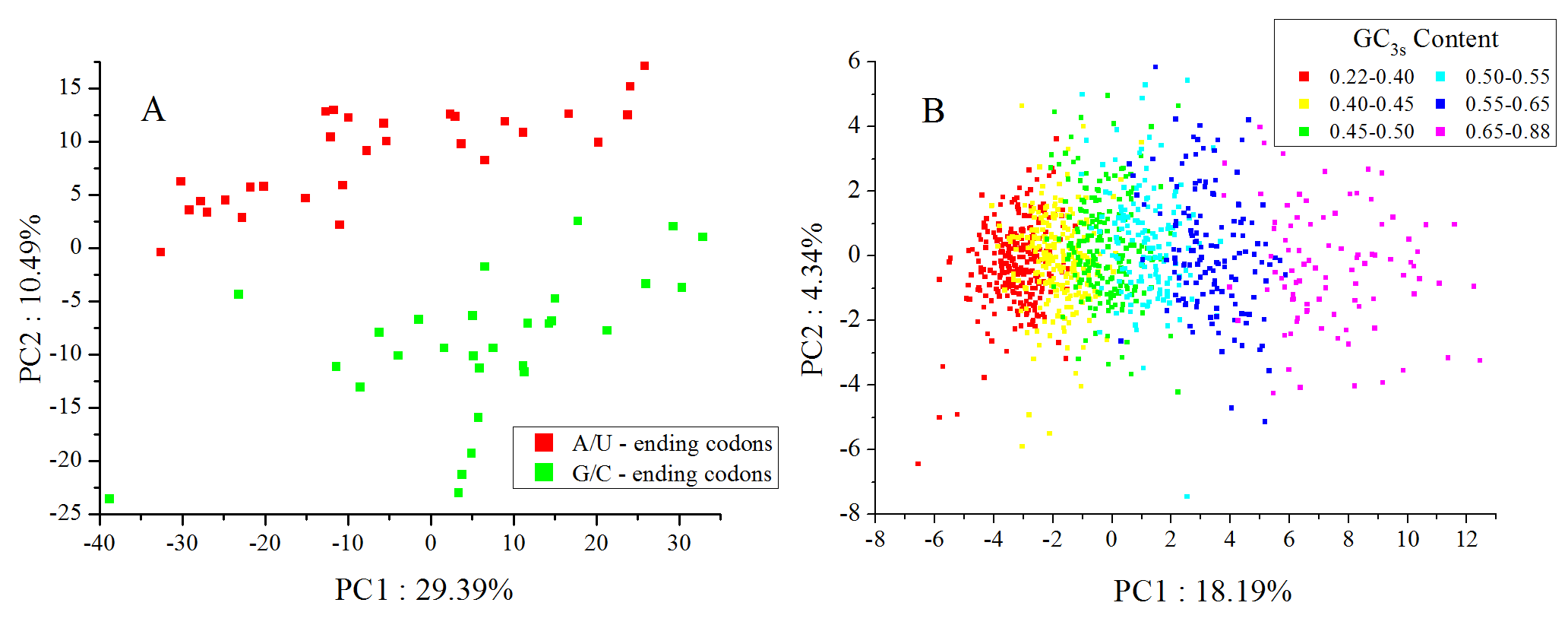

Supplement: Additional file 5 — PCA analysis of RSCU of codons within synonym groups from all bayberry ORF sequences. A) The distribution of 59 codons on the primary and secondary axes (accounting for 29.39% and 10.49% of the total variation, respectively), A/U-ending and G/C-ending codons are colored red and green, respectively. B) The distribution of 1,066 ORF sequences on the primary and secondary axes (accounting for 18.19% and 4.34% of the total variation, respectively), different groups of ORFs with GC3s content less than 0.40, 0.40 - 0.45, 0.45 - 0.50, 0.50 - 0.55, 0.55 - 0.65, and over 0.65, are colored red, yellow, green, cyan, blue and magenta, respectively. [file 1471-2164-14-732-S5.tiff]
